# Integrated analysis of co-expression, conserved genes and gene families reveal core regulatory network of heat stress response in *Cleistogenes songorica*, a xerophyte perennial desert plant

**DOI:** 10.1186/s12864-020-07122-8

**Published:** 2020-10-16

**Authors:** Qi Yan, Xifang Zong, Fan Wu, Jie Li, Tiantian Ma, Yufeng Zhao, Qian Ma, Penglei Wang, Yanrong Wang, Jiyu Zhang

**Affiliations:** State Key Laboratory of Grassland Agro-ecosystems, Key Laboratory of Grassland Livestock Industry Innovation, Engineering Research Center of Grassland Industry, Ministry of Education, College of Pastoral Agriculture Science and Technology, Lanzhou University, Lanzhou, 730020 People’s Republic of China

**Keywords:** Heat stress, *Cleistogenes songorica*, RNA sequencing, Conserved heat-responsive genes, Co-expression, Regulatory network, HSP, HSF

## Abstract

**Background:**

As global warming continues, heat stress (HS) is becoming an increasingly significant factor limiting plant growth and reproduction, especially for cool-season grass species. The objective of this study was to determine the transcriptional regulatory network of *Cleistogenes songorica* under HS via transcriptome profiling, identify of gene families and comparative analysis across major Poaceae species.

**Results:**

Physiological analysis revealed significantly decreased leaf relative water content (RWC) but increased proline (Pro) content in *C. songorica* under 24 h of HS. Transcriptome profiling indicated that 16,028 and 14,645 genes were differentially expressed in the shoots and roots of *C. songorica* under HS, respectively. Two subgenomes of *C. songorica* provide equal contribution under HS on the basis of the distribution and expression of differentially expressed genes (DEGs). Furthermore, 216 DEGs were identified as key evolutionarily conserved genes involved in the response to HS in *C. songorica* via comparative analysis with genes of four Poaceae species; these genes were involved in the ‘response to heat’ and ‘heat acclimation’. Notably, most of the conserved DEGs belonged to the heat-shock protein (HSP) superfamily. Similar results were also obtained from co-expression analysis. Interestingly, hub-genes of co-expression analysis were found to overlap with conserved genes, especially heat-shock protein (HSP). In *C. songorica*, 84 *HSP* and 32 heat-shock transcription factor (*HSF*) genes were identified in the allotetraploid *C. songorica* genome, and might have undergone purifying selection during evolutionary history based on syntenic and phylogenetic analysis. By analysing the expression patterns of the *CsHSP*s and *CsHSF*s, we found that the transcript abundance of 72.7% of the *CsHSP* genes and of 62.5% of the *CsHSF* genes changed under heat stress in both the shoots and roots. Finally, a core regulatory network of HS was constructed on the basis of the *CsHSP*, *CsHSF* and other responsive genes in *C. songorica*.

**Conclusions:**

Regulatory network and key genes were comprehensively analysed and identified in *C. songorica* under HS. This study improves our knowledge of thermotolerance mechanisms in native grasses, and also provides candidate genes for potential applications in the genetic improvement of grasses.

## Background

It has been widely confirmed that heat stress (HS) is one of the major limiting factors for plant growth and productivity worldwide [1]. Global temperature is likely to rise by 3–6 °C in the twenty-first century because of global warming, and the duration, intensity and frequency of high-temperature periods will increase during the vegetative stages and reproductive periods of plants [2, 3]. Therefore, it is important to determine the genomic, transcriptomic and physiological mechanisms of heat tolerance in plants, which are of great importance to accelerate crops and forages breeding aiming at thermotolerance improvement. *Cleistogenes songorica*, a C4 plant, is a desert grass that distributed widely in the wild lands in the northwest part of China [4]. *C. songorica* can grow in semi-arid, arid and desert regions where the annual rainfall ranges from 100 to150 mm. It is known to have novel drought stress adaptation strategies and contains rich pools of stress tolerance genes based on transcriptome profiling and identification of transcription factor and protein family [5–9]. Increasing amounts of evidence suggested that stress tolerance genes of native grass could facilitate tolerance improvement of plant for drought, heat, salt stress. For example, overexpression of the *heat-shock transcription factors* 1 (*HSFs*) of resurrection plant *Boea hygrometrica* leads to increased thermotolerance and retarded growth in transgenic *Arabidopsis* and tobacco [10]. Co-overexpression of *ZxNHX* and *ZxVP1–1* genes from *Zygophyllum xanthoxylum* enhances enhanced salinity and drought tolerance in *Lotus corniculatus* [11]. In previous study, we also confirmed that overexpression of functional genes form *C. songorica* also improved tolerance to drought and salt in both alfalfa and *Arabidopsis*, such as *CsLEA* and *CsALDH* [6–8]. However, the transcriptional regulatory network and potential candidate genes involved in HS response of native plant is not well documented, especially those of grass species.

Some studies have suggested that plants have developed complex regulatory networks at the biochemical, physiological and transcriptional levels to cope with HS; (1) The first response of plants to HS involves an increase in membrane fluidity of the plasmalemma. (2) This leads to the production of secondary messengers and chemical signals such as calcium in the cytoplasm. Mitogen-activated protein kinases (MAPKs) and calcium-dependent protein kinases (CDPKs) are in turn immediately regulated by the Ca^2+^ influx. (3) To maintain the water balance and osmotic adjustment in the cell, these signalling components are further transmitted to the nucleus, (4) resulting in the induction of antioxidants, electron transport and solutes that function osmotically in the cytoplasm, such as ascorbic acid, proline (Pro) and glutathione [12].

Along with the rapid development of sequencing technology, transcriptomic analysis is an extremely powerful tool for investigating plant molecular responses to abiotic stress, the knowledge of which could be further used to improve plant tolerance through molecular engineering. It has been widely used to identify heat-responsive genes in model plants, moss species, crop species and turfgrass species, such as *Arabidopsis* [13], rice [14], pepper (*Capsicum annuum* L.) [15], *Physcomitrella patens* [16], and *Agrostis scabra* [17]. Many HS-responsive genes have been identified in transcriptional regulatory network of model plant, including heat shock proteins (*HSPs*) and *HSFs* [18, 19]. *HSPs* play a key role in signal transduction and gene regulation under HS and can also interact with other stress response mechanisms [20]. HSPs protein could be divided into different groups on the basis of their molecular weight: for example, there are small HSP (sHSP), HSP70, HSP90 and HSP100 groups [21]. *HSP*s have been identified in plant species such as *Arabidopsis*, rice, *Brachypodium distachyon* and *Zea mays*, and the functions of these *HSP*s have been determined [21–23]. For instance, overexpression of *OsHSP26* can improve the tolerance of HS by protecting the photosystems [24]. Furthermore, *HSP*s can regulate the expression of *HSF*s, which largely participate in gene regulation under HS. HSF transcription factor family can be divided into A, B and C groups and play important roles in signalling pathways and transcriptional regulation [25]. For example, as compared with wild-type plants, transgenic *AtHsfA1* plants are significantly more tolerant to heat and drought stress [26]. *HSP70*/*HSP90* decreases the inhibition of *HSFA1*s under HS, resulting in the activation or reduction in gene expression by downstream transcription factors (TFs). Similarly, mutant *Arabidopsis HSFA1* plants are sensitive to HS [27]. *Dehydration-responsive element binding protein 2A* (*DREB2A*), a direct target gene of *HSFA1*, is a key gene involved in post-translational regulation under HS. *DREB2A* can positively regulate *HSFA3* gene expression by interacting with other genes, such as the subunits of *nuclear factor Y A2* (*NF-YA2*), *NF-YB3* and *DNA polymerase II subunit B3–1* (*DPB3–1*) [28, 29]. *HSFA3* can activate the expression of downstream genes, including those encoding chaperones and enzymes. Overexpression or mutation of *HSFA3* in *A. thaliana* was shown to increase or decrease the tolerance of transgenic plants, respectively [29, 30]. In addition, *HSFA2* and *HSFA7* act as target genes of *HSFA1*, which involves two adjustment processes: *HSFA2* and *HSFA7* can be directly positively regulated by *HSFA1*, and *HSFA1* negatively regulates *HSFA2* and *HSFA7* by inducing the expression of *HSFB*. Strikingly, *HSFB* not only is controlled by *HSFA1* but also inhibits the activity of *HSFA1* via a negative feedback loop [18].

In the present study, we performed a genome-wide transcriptomic analysis of *C. songorica*, comparative analysis across major Poaceae species, co-expression network, and identification of gene families under HS. We aimed to address the following main questions: (i) How many/which genes exhibit significant changes in expression in *C. songorica* under stress? (ii) What key Gene Ontology (GO) terms and Kyoto Encyclopedia of Genes and Genomes (KEGG) pathways are enriched? (iii) How many/which genes play key roles and are conserved or co-expression under HS in the Poaceae? Our study provided new insights into the regulatory mechanism of *C. songorica* under HS and identified candidate genes associated with HS, these genes may be used for molecular breeding new varieties with thermotolerance.

## Results

### Phenotype and physiology responses of *C. songorica* under HS

To study the physiological changes caused by HS, nine-week-old seedlings of *C. songorica* were placed in three growth chamber with a 40 °C temperature during the day and night for 0 h, 6 h, 12 h, 24 h, 36 h, 48 h or 72 h. Under high-temperature exposure, the relative water content (RWC) gradually decreased and significantly differed (*p* < 0.05) at 24 h and a few leaves began to curl in *C. songorica* (Additional file 1: Fig. S1a-b). Remarkably, compared with that at 0 h, the RWC after 48 h of treatment decreased by 36.4%. Under high temperature treatment, leaf temperature was stably increased with decreasing water content, whereas soil temperature increased more rapidly in *C. songorica* (Additional file 1: Fig. S1h-i). Leaf and soil temperature increased by 1.35- and 1.15-fold at 6 h treatment, respectively. Additionally, the Pro and malondialdehyde (MDA) contents gradually increased under HS and markedly increased at 6 h and 48 h, respectively, in *C. songorica* seedlings; compared with those at 0 h, the Pro and MDA contents increased by 14.9- and 1.46-fold, respectively (Additional file 1: Fig. S1c-d). Moreover, we also analysed the changes in photosynthesis traits under HS. The results indicated that injury to the photosystems occurred very quickly under HS. The Fv/Fm, rETRmax and quantum yield of photosystem II (PSII (Y(II)) significantly decreased (*p* < 0.05) to 55.96, 64.25 and 60.07% of their respective values at 0 h, respectively, after 6 h of treatment (Additional file 1: Fig. S1e-g).

### Identification of DEGs in the subgenome of *C. songorica*

**Fig. 1:**
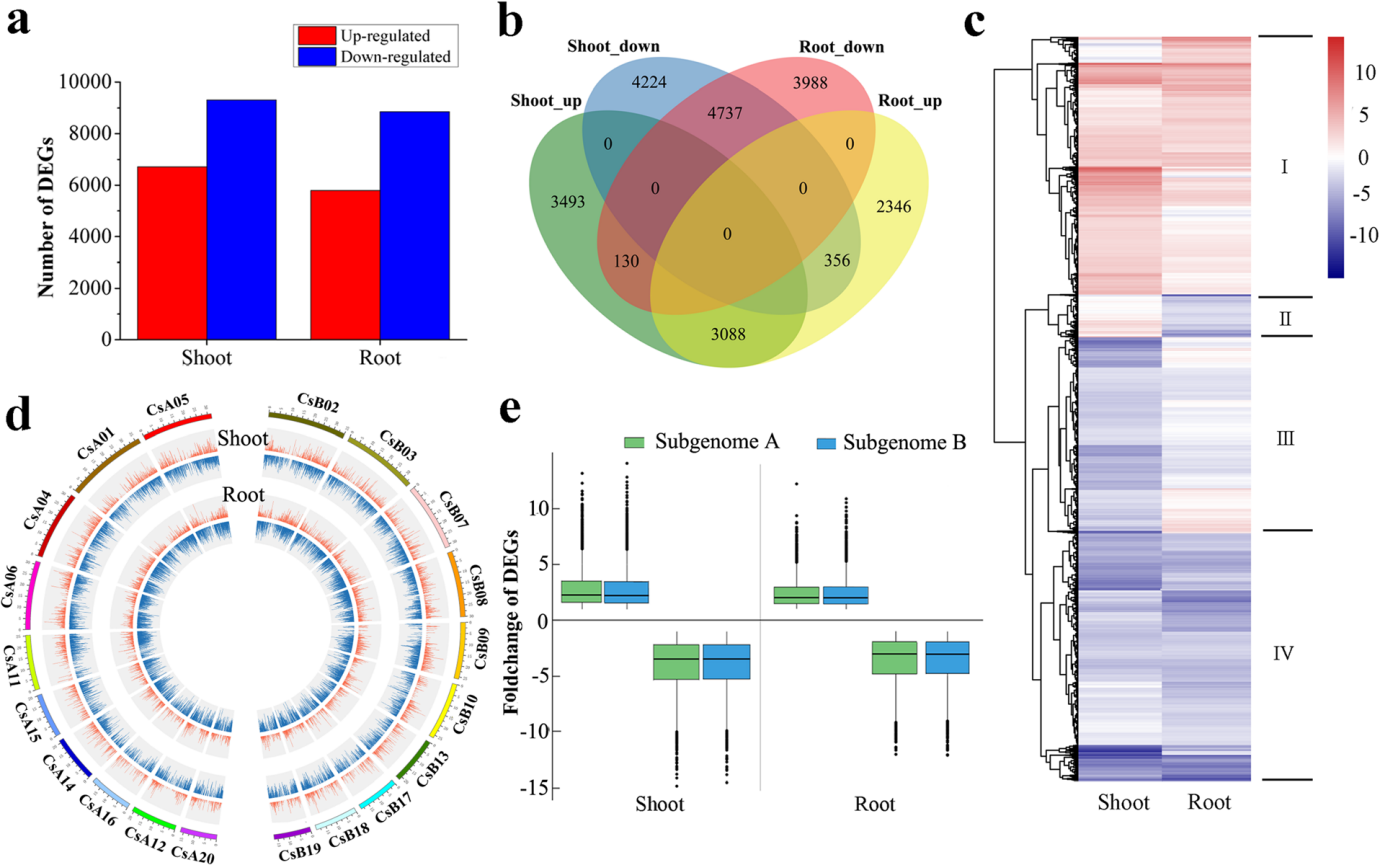
Identification and characteristics of DEGs in *C. songorica* under HS. **a** Number of DEGs (fold change ≥2; FDR ≤ 0.01) in the shoots and roots. **b** Venn diagram indicating the overlapping DEGs between the shoots and roots. **c** Cluster analysis of DEGs in *C. songorica* under HS. **d** Circos chart showing the location and log_2_(fold change) of DEGs within two subgenomes of *C. songorica*. The orange and blue lines represent the upregulated and downregulated DEGs, respectively. **e** Box plot indicating log_2_ (fold change) of DEGs from different subgenomes of *C. songorica* in the roots and shoots

To reveal the changes in transcript levels in the roots and shoots of *C. songorica* under HS, samples collected at 0 h and 24 h were subjected to genome-wide RNA sequencing (RNA-seq) analysis. A total of 657,826,480 clean reads from 12 samples (two treatments, two tissues, three biological replicates) were mapped to the *C. songorica* genome after filtering and quality control were performed. The Q30 contents and mapping percentages were mostly greater than 91 and 80%, respectively (Additional file 2: Table S1). To validate the RNA-seq data, nine genes were chosen for qRT-PCR, including *HSFA1–2*, *HSFA3–1*, *HSFA3–2*, *HSFA9–1*, *HSFA9–2*, *HSP90–6*, *HSP70–7*, *JUB1* and *NF-YB3–2*. The results indicated that the expression profiles of these gene were coincident with gene expression of RNA-seq (Fig. 9b). In total, 22,362 genes were significantly differentially expressed under HS (log2(fold change) ≥ 1; FDR ≤ 0.01) in the roots and shoots. Among these genes, the number of DEGs in the shoots was greater than that in the roots (16,028 and 14,645 DEGs, respectively; Fig. 1a; Additional file 3: Table S2). Strikingly, the numbers of upregulated and downregulated DEGs were significantly different. For example, there were 6711 upregulated DEGs and 9317 downregulated DEGs in the *C. songorica* shoots. A similar trend was also found in the roots. By comparing the 8311 DEGs common to the different organs, we observed a further general conservation of expression, with the expression of 7825 of the genes being regulated in the same way in the shoots and roots and that of 486 genes displaying opposite regulatory patterns (Fig. 1b). A total of 4737 downregulated and 3088 upregulated DEGs were found both in shoots and roots. As shown in Fig. 1c, the gene expression pattern was obviously changed under HS and DEGs could be grouped into four expression clusters; cluster I and cluster IV consisted of DEGs whose expressions were upregulated and downregulated both in the shoots and roots, respectively (Fig. 1c).

*C. songorica* is an allotetraploid plant, so we also analysed the distribution of DEGs in the two subgenomes of *C. songorica*. The results indicated that the DEGs were uniformly distributed in the two subgenomes of *C. songorica*. A detailed assessment of the numbers of DEGs between the subgenome A and subgenome B conditions for each organ confirmed this observation; in the shoots, there were 2716 DEGs whose expression was upregulated (4255 downregulated DEGs) and 2874 DEGs whose expression was upregulated (4298 downregulated DEGs) in subgenome A and subgenome B, respectively (Fig. 1d; Additional file 3: Table S2). Similar trends were identified in the roots of *C. songorica*. We also analysed the expression levels of the DEGs on the basis of fold changes and found that the expression of the DEGs was similar between subgenome A and subgenome B of *C. songorica* (Fig. 1e).

### GO enrichment analysis of DEGs

**Fig. 2:**
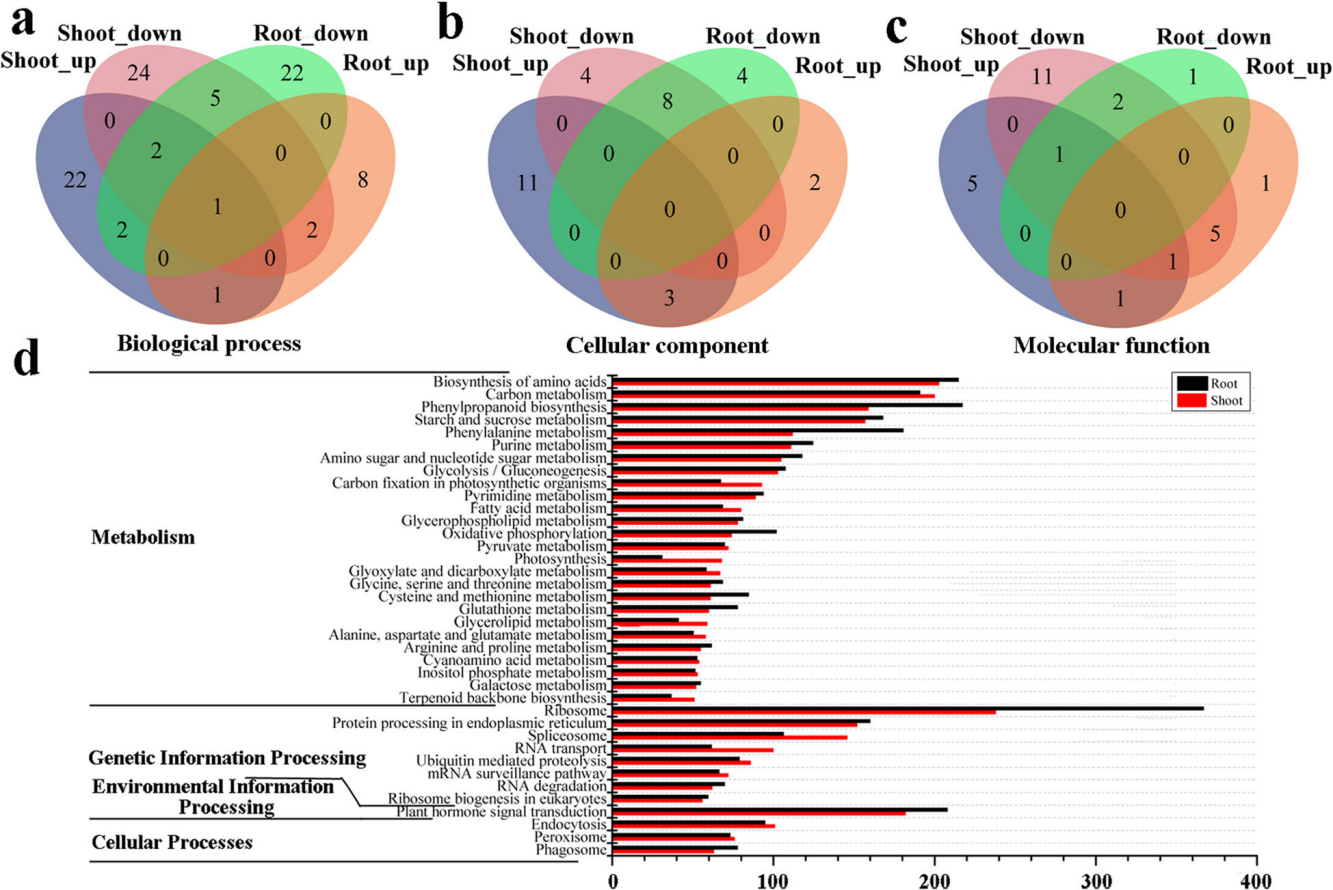
Functional analysis of DEGs in *C. songorica* under HS. Venn diagram of significantly enriched GO terms. GO terms that were over-represented under different conditions. **a** Biological process; **b** cellular component; **c** molecular function. *Shoot_down*: DEGs whose expression was downregulated in the shoots; *Shoot_up*: DEGs whose expression was upregulated in the shoots; *Root_down*: DEGs whose expression was downregulated in the roots; *Root_up*: DEGs whose expression was upregulated in the roots; **d** Distribution of KEGG pathways for DEGs in the roots and shoots. Only categories with more than 20 DEGs in roots are shown. *Red bar*, DEGs in the shoots; *black bar*, DEGs in the roots

To identify the major functions of the heat-responsive genes in *C. songorica*, we performed a GO enrichment analysis of the DEGs in the shoots and roots of *C. songorica*. On the basis of the GO enrichment results, we found that the majority of DEGs were involved in stress-related categories, including ‘response to stimulus’, ‘nucleic acid binding transcription factor activity’, ‘organelle’, and ‘developmental process’. Notably, the biological process category revealed differences in the number of associated DEGs between the two organs, with greater expression in the shoots than in the roots (Additional file 1: Fig. S2). To identify the functional enrichment categories of the DEGs further, we used a *p*-value< 0.05 to filter the significantly enriched GO categories. We also compared the numbers of GO categories between the different organs and the direction of regulation. Four comparisons were performed: shoot_up (upregulated genes in the shoots), shoot_down (downregulated genes in the shoots), root_up (upregulated genes in the roots) and root_down (downregulated genes in the roots) (Fig. 2).

One biological process GO term was found in the shoot and root comparisons, while no cellular component or molecular function GO terms were revealed (Fig. 2; Additional file 4: Table S3). The specific shoot_down- and shoot_up-associated GO terms were related mainly to photosynthesis (‘photosystem II assembly’, ‘photosynthesis, light reaction’, ‘chlorophyll biosynthetic process’, ‘chloroplast organization’) and the stimulus response (‘response to heat’, ‘cellular response to endogenous stimulus’), respectively, in the biological process category (Fig. 2a; Additional file 4: Table S3). Notably, root_down and root_up were associated mainly with morphogenesis (‘cell morphogenesis’, ‘mitotic cell cycle process’, ‘developmental growth involved in morphogenesis’) and phosphorylation (‘phosphorylation’, ‘protein phosphorylation’), respectively (Fig. 2a; Additional file 4: Table S3). In the molecular function category, there were eight common GO terms between the shoot_down and root_down groups, which were related to many enzyme activities (‘peroxidase activity’, ‘hydrolase activity’; Fig. 2c; Additional file 4: Table S3). In the cellular component category, the shoot_down-specific terms were similar to the biological process terms and were associated with the chloroplast (‘chloroplast thylakoid’, ‘chloroplast’). The shoot_down and root_up groups presented five common terms, which were also related to the chloroplast (‘chloroplast thylakoid membrane’, ‘chloroplast envelope’, ‘chloroplast stroma’; Fig. 2b; Additional file 4: Table S3).

### Key KEGG pathways of DEGs

We investigated the key metabolic pathways that respond to HS in *C. songorica*. The results revealed several major metabolic pathways among the stress responses. The ‘biosynthesis of amino acids’ and ‘ribosome’ pathways were the most significant pathways involved in metabolism and genetic information processing, respectively (Fig. 2d). Interestingly, the numbers of DEGs associated with ‘ribosome’ significantly differed between the organs, with a 1.5-fold greater number in the roots than in the shoots. Two pathways related to phenylpropanoids, ‘phenylpropanoid biosynthesis’ and ‘phenylalanine metabolism’, were significantly represented under HS and presented a greater number of DEGs in the roots than in the shoots. Furthermore, several DEGs were involved in energy metabolism, and their expression was downregulated under HS; these DEGs included those associated with the ‘starch and sucrose’, ‘amino sugar and nucleotide sugar metabolism’ and ‘glycolysis/gluconeogenesis’ categories (Fig. 2d). The expression of most of the DEGs involved in the photosynthesis pathway was also downregulated in *C. songorica* under HS, such as those in the ‘carbon fixation in photosynthesis organisms’ and ‘photosynthesis’ categories. With respect to genetic information processing, the expression of many DEGs related to ‘RNA transport’ and the ‘spliceosome’ was upregulated in *C. songorica*. In addition, two pathways presented more DEGs in the shoots than in the roots. DEGs in several other metabolic and signal transduction pathways, such as ‘arginine and proline metabolism’, ‘pyrimidine metabolism’, ‘fatty acid metabolism’ and ‘plant hormone signal transduction’ pathways, were also found to be differentially expressed in *C. songorica* (Fig. 2d).

### Differential expression of TF-encoding genes

We also identified DEGs encoding members of TF families in *C. songorica*. In total, the genes encoding 1692 TFs belonging to 50 families were differentially expressed under HS in *C. songorica* (Additional file 5: Table S4). In the shoots and roots, 1692 and 1517 TFs, respectively, were identified among the DEGs. Strikingly, the number of downregulated TF genes was greater than the number of upregulated TF genes in the shoots and roots (Additional file 1: Fig. S3). In subgenome A and subgenome B of *C. songorica*, the distribution of regulated TF genes was similar. Furthermore, we found that the numbers of upregulated and downregulated genes that encode TFs were greater in subgenome B than in subgenome A in the roots. The number of downregulated TF genes in subgenome B was greater than that in subgenome A only in the shoots (Additional file 1: Fig. S3b). The greatest number of TFs belonged to the Nin-like family, followed by the MYB and NAC families (Table 1; Additional file 1: Fig. S3c). Furthermore, the numbers of several TF families significantly differed between the shoots and roots. The numbers of DEGs in the Nin-like, MYB and C3H families were greater in the shoots than in the roots (Table 1; Additional file 1: Fig. S3c). The number of DEGs encoding bHLH family members was lower in the shoots than in the roots. We observed that 23 HSF members in *C. songorica* were homologous to 17 HSF TFs in rice. Furthermore, seven NF-YAs and seven NF-YBs TFs were identified in DEGs, respectively (Additional file 5: Table S4). The numbers of DEGs of several families, such as the NAC, ERF, bZIP, and WRKY families, were the same between the shoots and roots (Table 1). Overall, these results suggested that the regulation of TFs played a crucial role in the response of *C. songorica* to HS.

**Table 1:** Distribution of transcription factors responsive to heat stress in *C. songorica*

Transcription factors family	Transcription factors in *C.songorica* shoot DEGs	Transcription factors in *C.songorica* root DEGs
Nin-like	169	121
MYB	157	126
NAC	117	120
ERF	106	105
bHLH	105	122
C2H2	98	81
C3H	84	58
bZIP	82	77
B3	78	78
WRKY	64	68
FAR1	61	57
G2-like	59	43
ARF	50	47
HD-ZIP	48	36
NF-YC	36	36
CO-like	32	20
Trihelix	29	24
GRAS	27	27
HSF	23	19

Note: Only categories with more than 20 DEGs identified as transcription factors are shown

### Identification of a core set of Poaceae genes that are differentially regulated under HS

**Fig. 3:**
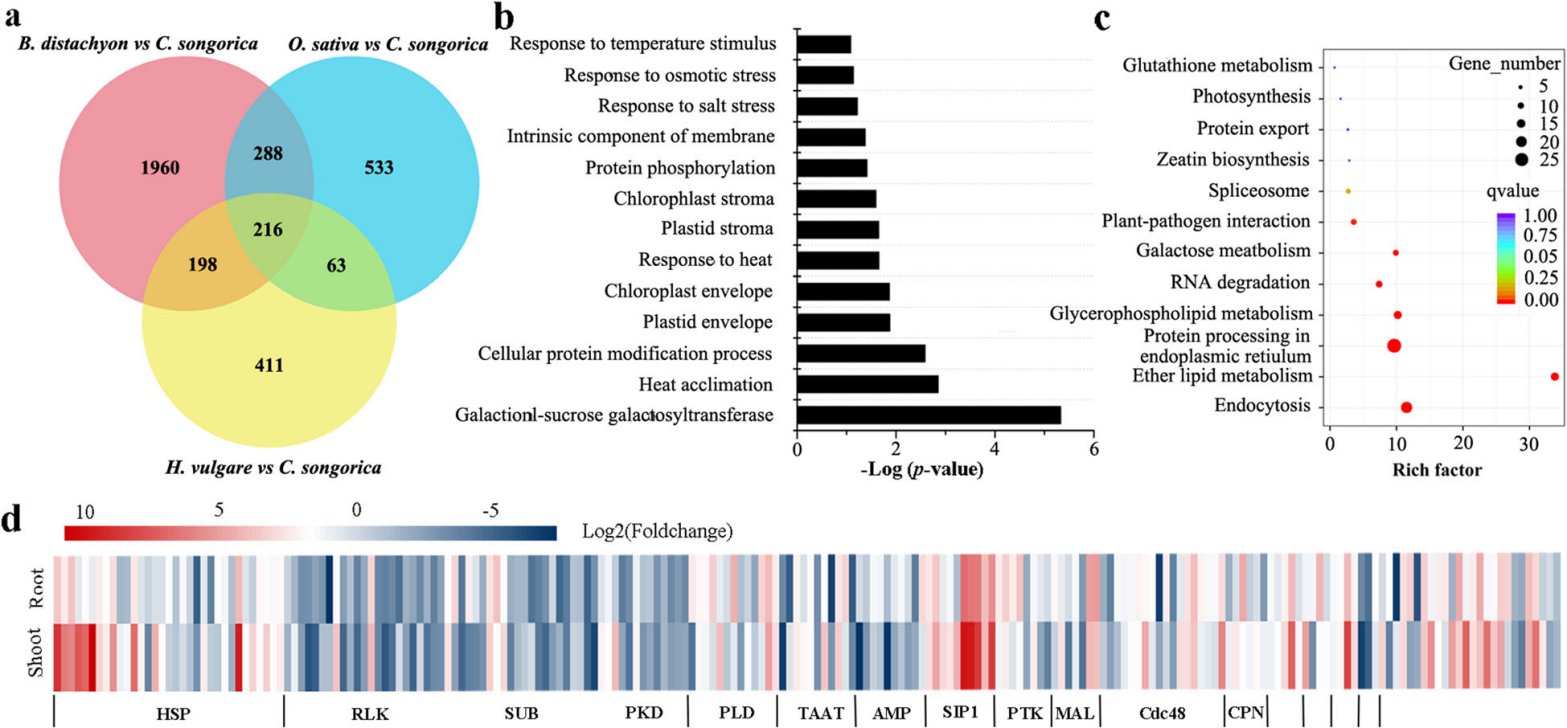
Comparison of conserved DEGs that respond to heat across *C. songorica*, rice, *B. distachyon* and *H. vulgare*. **a** Venn diagram showing putative orthologues of *C. songorica* stress-responsive genes identified by OrthoMCL in at least two species. **b** Significantly enriched GO terms of conserved DEGs. **c** KEGG enrichment analysis of conserved DEGs. **d** Heat map showing the log_2_(fold change) of key gene families among the conserved DEGs in shoot and root. HSP: heat-shock protein; RLK: leucine-rich repeat receptor-like kinases; SUB: subtilase family; PKD: protein kinase domain; PLD: phospholipase D; TAAT: transmembrane amino acid transporter protein; SIP1: raffinose synthase/seed imbibition protein; PTK: protein tyrosine kinase; CPN: TCP-1/cpn60 chaperonin family; MAL: Malectin domain; Cdc48: Cdc48 subfamily

To identify the functionally conserved genes in different species, we used four Poaceae species to identify the subsets of HS-responsive DEGs. In addition to these heat-responsive DEGs, we identified 1120, 888 and 2662 orthologues from rice, *Hordeum vulgare* and *B. distachyon*, respectively (Fig. 3a; Additional file 6: Table S5). In total, 216 orthologues that played a key role in the response to HS during evolution were identified in all the studied species (Fig. 3a; Additional file 6: Table S5). The GO enrichment analysis revealed that the conserved DEGs were enriched in ‘stress responses’ and ‘photosynthesis’. For example, 37 and 41 conserved genes were enriched in the ‘response to heat’ and ‘response to temperature stimulus’, respectively. Furthermore, 12 conserved DEGs were involved in ‘heat acclimation’, and another 12 were involved in the ‘response to osmotic stress’ (Fig. 3b). Several other conserved DEGs were associated with chloroplasts; for example, 16 and 13 conserved DEGs were enriched in the ‘chloroplast envelope’ and ‘chloroplast stroma’, respectively. According to the prediction of the gene families via the Pfam database, a total of 31 gene families were identified. The largest family was the HSP family (15.3%), followed by the leucine-rich repeat receptor-like kinases (RLK) protein family (10.6%), the subtilase family (9.7%), the protein tyrosine kinase (PTK) superfamily (6.5%), the phospholipase D family (PLD, 6%), and the raffinose synthase/seed imbibition protein family (SIP1, 4.6%; Fig. 3d).

The KEGG pathway analysis revealed that some conserved DEGs were involved in the biosynthesis of different metabolites, including lipids and sugars (Fig. 3c). The conserved DEGs were most significantly enriched in ‘ether lipid metabolism’. The expression of 12 and 2 putative *PLD* genes was upregulated and downregulated in *C. songorica*, respectively (Additional file 6: Table S5). In terms of sugar metabolism, 10 conserved DEGs were annotated as raffinose synthase/seed imbibition protein, and the expression of these was upregulated in the shoots and roots. In addition to glutathione metabolism, the expression of *glutathione s-transferase* (*GST*) was downregulated in *C. songorica*. Remarkably, ‘protein processing in endoplasmic reticulum’ included 7 *HSP* genes whose expression was upregulated in *C. songorica* under HS. Moreover, among the conserved DEGs, the *photosystem II subunit R* (*PsbR*) gene was identified, and its expression was upregulated (Additional file 6: Table S5).

KOG function classification indicated that these conserved DEGs involved in ‘postertranslational medication, protein turnover, chaperones’, ‘signal transduction mechanisms’ and lipid transport and metabolism’ (Additional file 1: Fig. S4). The genes of RLK family were identified in the class of ‘signal transduction mechanisms’, most of which were downregulated in shoot and root under HS.

### Analysis of gene co-expression

In order to measure the result of conserved responsive genes and identify the hub genes under HS, the co-expression network analysis was performed using the WGCNA package. The DEGs with low abundance (FPKM≤1) and low variability were filtered out in order to reduce noise. As a result of weighted co-expression network analysis, a module that was positively correlated with HS was identified and included 297 DEGs (Additional file 7: Table S6). The result of GO annotation showed that co-expressed DEGs involved stress responses (eg. ‘response to stress’ (sixty two DEGs), ‘response to abiotic stimulus’ (thirty five DEGs), ‘response to temperature stimulus’ (sixteen DEGs), ‘response to heat’ (six DEGs)), signalling (eg. ‘signaling’ (twenty one DEGs), ‘signal transduction’ (twenty one DEGs), ‘hormone-mediated signaling pathway’ (fourteen DEGs)) and regulations (eg. ‘regulation of biosynthetic process’ (thirty six DEGs), ‘regulation of gene expression’ (thirty four DEGs), ‘regulation of response to stimulus’ (nine DEGs) (Additional file 8: Table S7). Co-expressed DEGs were enriched in ‘plant hormone signal transduction’, ‘plant-pathogen metabolism’, and ‘starch and sucrose metabolism’ based on KEGG enrichment analyses (Additional file 1: Fig. S5).

**Fig. 4:**
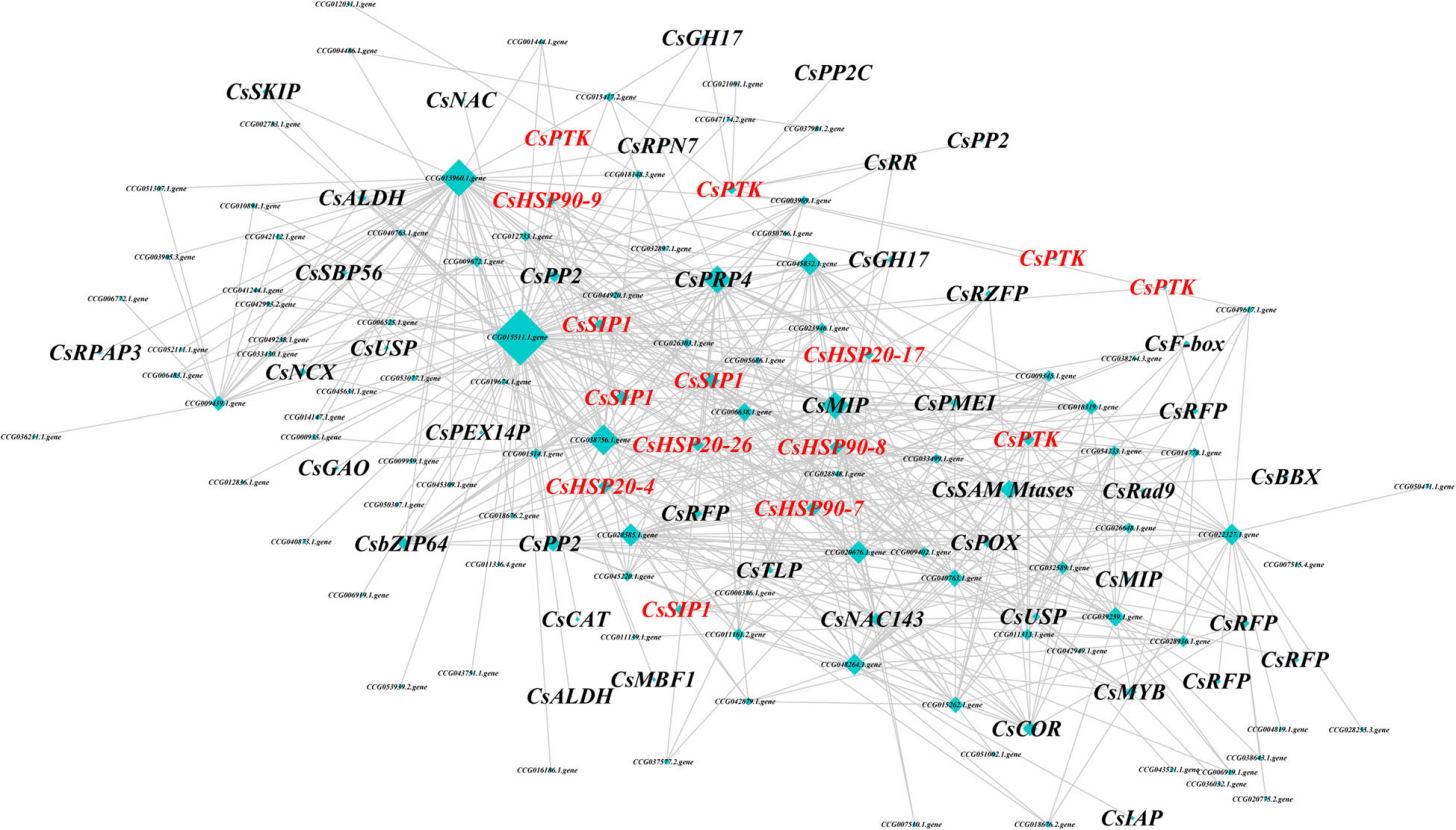
Co-expression analyses of *C. songorica* under HS. Co-expression gene networks with the greatest hubness. Nodes and edges are represented by squares and lines coated with color, respectively. The bigger the squares, the higher the more edges. CsPRN7: 26S proteasome subunit RPN7; CsSBP56: 56 kDa selenium binding protein; CsALDH; Aldehyde dehydrogenase; CsBZIP: Basic region leucine zipper; CsCOR: Cold acclimation protein; Cs F-box: F-box protein; CsGH17: Glycosyl hydrolases family 17; CsHSP20: Hsp20 protein; CsHSP90: Hsp90 protein; CsIAP: Inhibitor of apoptosis-promoting; CsMIP: Major intrinsic protein; CsMBF: Multiprotein bridging factor 1; CsMYB: Myb transcription factor; CsNAC: No apical meristem (NAM) protein; CsRPAP3; Potential Monad-binding region of RPAP3; CsPTK; Protein tyrosine kinase; CsPP2C: Protein phosphatase 2C; CsPRP4: pre-mRNA processing factor 4; CsSAM-Mtases: S-adenosyl-L-methionine-dependent methyltransferase; CsRad9: Rad9; CsSIP1: Raffinose synthase or seed imbibition protein; CsRFP: Ring finger protein; CsSBP: Squamosa promoter-binding-like protein; CsNCX; Sodium/calcium exchanger protein; CsTLP: Thaumatin-like protein; CsUSP: Universal stress protein; CsPP2: Phloem protein 2; CsPOX; Peroxidase; CsPEX14: Peroxisomal membrane protein; CsSKIP: SNW/SKI-interacting protein; CsBBX: B-box zinc finger protein; CsPMEI: Plant invertase/pectin methylesterase inhibitor; CsRR: response regulator-like PRR1; CsGAO: Galactose oxidase; CsCAT: Catalase

In order to view the detailed expression patterns of pivotal individual genes, we assigned hub genes in co-expression network. The edge of co-expression network with low weight value (weight value≤0.57) were filtered out in order to reduce noise. Totally, 582 co-expression relationships and 152 DEGs were found in co-expression network under HS (Fig. 4, Additional file 9: Table S8). Based on the result of gene annotation, we found that the conserved heat-responsive DEGs of previous results were also identified in co-expression network, including six *CsHSPs*, five *CsPTKs* and four *CsSIPs*. This results suggested these conserved genes played a core role in regulatory networks of *C. songorica* under HS, and confirmed that our analysis results were reliable to reflect the regulatory networks of *C. songorica* under HS. In addition, we also found four TFs, including two *CsNAC*, one *CsBZIP* and one *CsMYB* genes (Fig. 4; Additional file 9: Table S8). In ‘plant hormone signal transduction pathway’, we found that one *CsPP2C*, four Cs*PTKs*, one cold acclimation protein gene (*CsCOR*) were significantly induced under HS. The one peroxisomal membrane protein (*CsPEX*), one 56 kDa selenium binding protein (*CsSBP56*) and one sodium/calcium exchanger protein (*CsNCX*) genes were annotated in ‘inorganic ion transport and metabolism’ pathway. In addition, two major intrinsic protein genes (*CsMIP*) belonged to aquaporin TIP2 and aquaporin SIP1, respectively (Fig. 4; Additional file 9: Table S8). Multiprotein bridging factor 1 (*CsMBF*), a key regulated gene of HSFA1, were also identified in co-expression network. Other abiotic stress responsive genes were also identified in co-expression network, such as aldehyde dehydrogenase genes (*CsALDH*), Universal stress protein gene (*CsUSP*), RING- finger protein genes (*CsRFP*), Peroxidase (*CsPOX*), Catalase (*CsCAT*). Interestingly, some key hub genes belonged to unknown function family and didn’t have gene annotation, such as CCG015511.1.gene, CCG015960.1.gene, CCG038756.1.gene, and CCG032589.1.gene (Fig. 4; Additional file 9: Table S8).

### Identification and analysis of *HSP* and *HSF* superfamily members in *C. songorica*

**Fig. 5:**
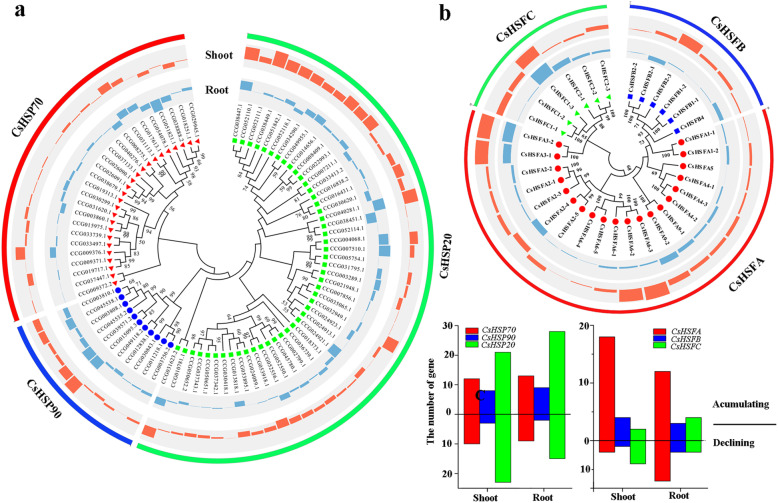
Identification and expression patterns of *HSP* and *HSF* gene families in *C. songorica* during HS. **a** Neighbour-joining tree showing the phylogenetic relationships between 84 HSP proteins and a histogram showing the log_2_(fold change) of the *HSP* genes in the shoots and roots under HS. **b** Neighbour-joining tree showing the phylogenetic relationship between 32 HSF proteins and a histogram showing the log_2_(fold change) of the *HSF* genes in the shoots and roots under HS. The dots on the branches represent bootstrap support values. **c** Numbers of upregulated and downregulated *HSP* and *HSF* genes in the shoots and roots under HS

Our results suggested that the HSPs and HSFs played core roles in HS response of *C. songorica*. Therefore, the *HSP* and *HSF* superfamily of *C. songorica* were identified and analysed. Totals of 48, 25, 11, 20, 6 and 6 genes in the *C. songorica* genome were confirmed to be *HSP20*, *HSP70*, *HSP90*, *HSFA*, *HSFB*, and *HSFC* genes, respectively, according to BLAST analysis (Fig. 5; Additional file 10: Table S9). These genes were named based on their chromosomal location and group. Analysis of the expression pattern revealed that 44 *CsHSP20* (91.7%), 22 *CsHSP70* (88%), 11 *CsHSP90* (100%), 20 *CsHSFA* (100%), 5 *CsHSFB* (88.3%) and 6 *CsHSFC* (100%) genes were expressed under HS in the shoots (Fig. 5c); the same result was found in the roots. The numbers of upregulated and downregulated *CsHSP70* genes were almost identical in the shoots and roots, but there were more upregulated *CsHSP90* and *CsHSFA* genes than downregulated ones in these groups in the shoots and roots. Remarkably, there were more upregulated than downregulated *CsHSB* genes in the shoots, but there were more upregulated than downregulated *CsHSP20* and *CsHSFC* genes in these groups in the roots (Fig. 5c). The RT-qPCR results showed that HS significantly increased the expression levels of *CsHSFA3–1*, *CsHSFA3–2*, *CsHSFA9–1*, *CsHSFA9–2* and *CsHSFA1–2* in *C. songorica*; however, the expression levels of *CsHSP70–7* and *CsHSP90–6* decreased under HS (Fig. 9b).

**Fig. 6:**
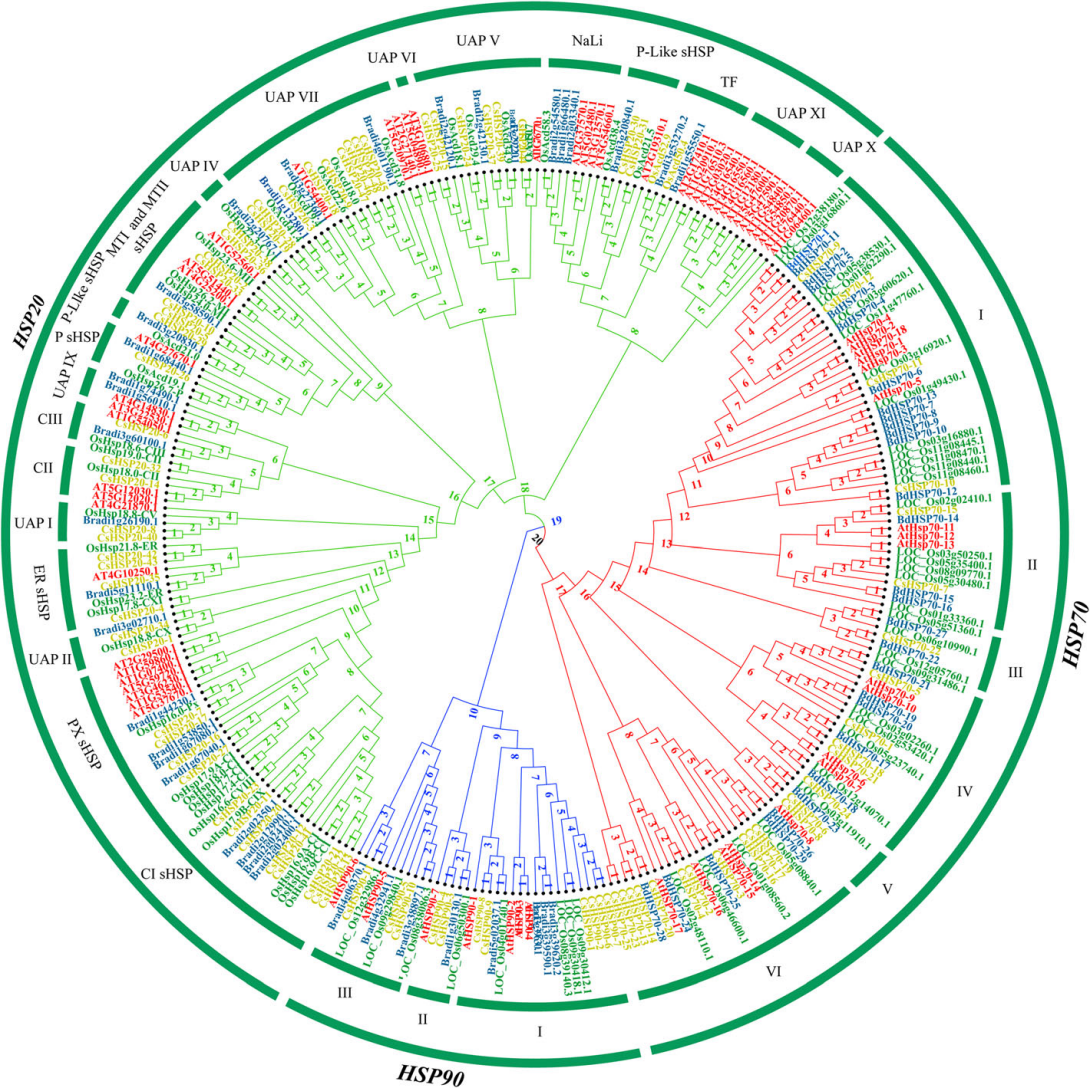
Phylogenetic tree of HSP proteins in four species: *C. songorica* (yellow), rice (green), *B. distachyon* (blue) and *Arabidopsis* (red). The tree was generated with IQTREE software via the ML method

To analyse their evolutionary relationships, the HSP and HSF proteins of *Arabidopsis*, rice, *B. distachyon* and *C. songorica* were used to construct an unrooted phylogenetic tree. In total, 299 HSP proteins and 126 HSF proteins were included in the phylogenetic tree (Fig. 6; Additional file 1: Fig. S6). The phylogenetic branch of the HSP proteins was divided into the HSP20, HSP70 and HSP90 families (Fig. 5a). The 34 HSP90 proteins were classified into three subfamilies, which were designated as subfamilies I, II and III (Fig. 5). Subfamily I of HSP90 contained the most CsHSP90 proteins (6 CsHSP90s, 54.5%). In addition, 104 HSP70 proteins were divided into 6 groups, designated as groups I, II, III, IV, V and VI. Among the six groups of HSP70 proteins, groups I and V were the largest (36 CsHSP70s, 34.6%) and smallest (5 CsHSP70s, 4.8%), respectively (Fig. 6). Notably, group III lacked HSP70 proteins from *Arabidopsis*. Moreover, the UAP XI, UAP X, and UAP VI subfamilies contained HSP20 proteins from only *Arabidopsis*, and the UAP V, UAP IV, UAP I and UAP II subfamilies were specific to Poaceae species (Fig. 6). The HSF proteins were divided into three subfamilies: HSFA, HSFB, and HSFC. The HSFA subfamily was the largest subfamily and contained nine groups (Fig. 5b). Notably, *CsHSF* was not grouped with *HSFA7*, *HSFA8*, or *HSFB3* (Additional file 1: Fig. S6).

### Genome synteny and variation analysis of the HSP and HSF families in *C. songorica*

**Fig. 7:**
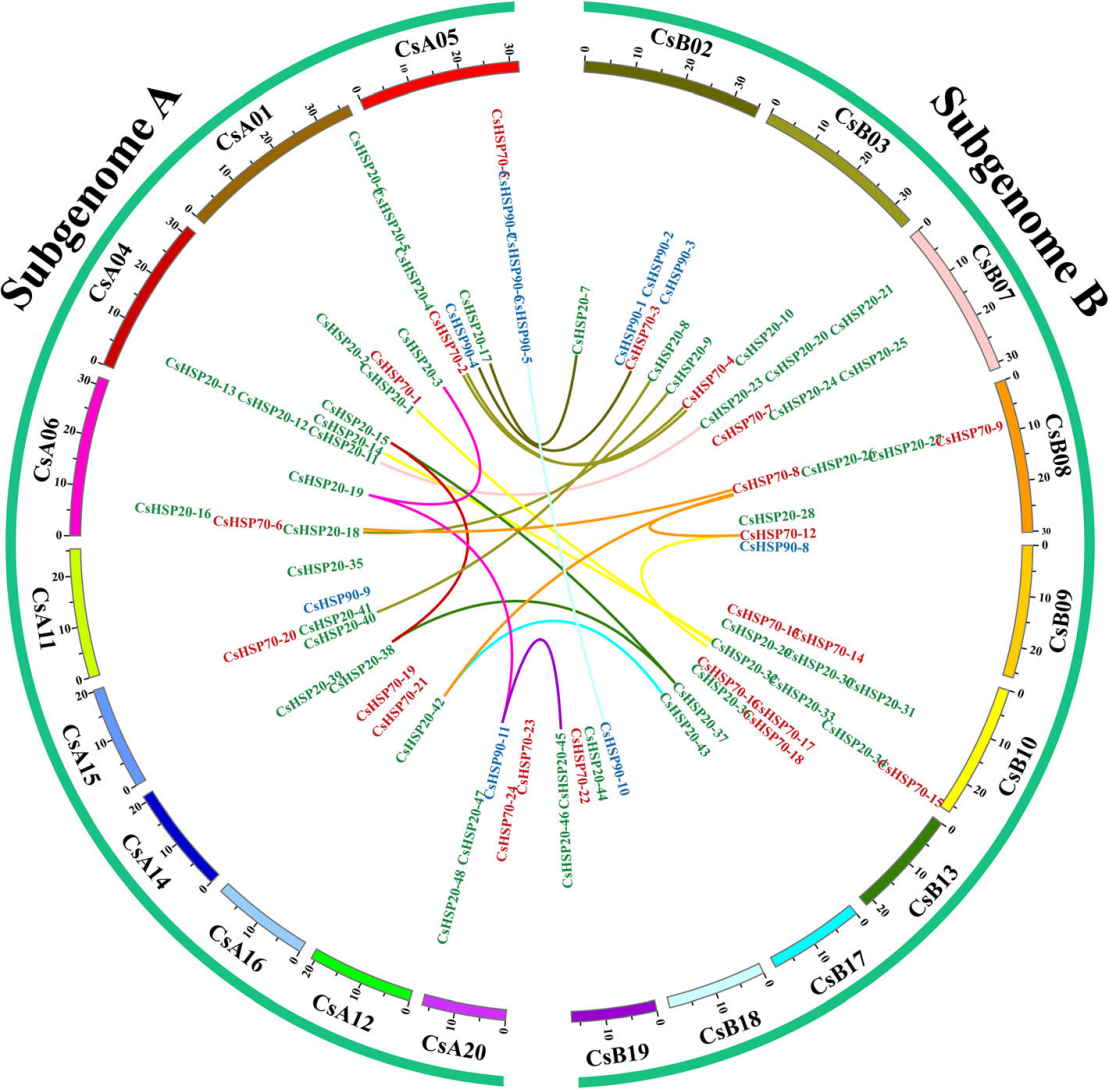
Distribution and synteny analysis of *C. songorica HSP* genes. The 20 *C. songorica* chromosomes are shown as different coloured partial circles, and the chromosome numbers are indicated at the top of each bar. The coloured links indicate *HSP* syntenic regions in the *C. songorica HSP* gene family. *HSP20*, green; *HSP70*, red; *HSP90*, blue

Totals of 83 *CsHSP* (98.8%) and 30 *CsHSF* (93.8%) genes were located on the chromosomes of *C. songorica*, and these genes were found to be randomly distributed (Fig. 7; Additional file 11: Table S10). These genes were also evenly distributed between the two subgenomes of *C. songorica*. CsB10, CsA01 and CsB07 contained the most *CsHSP20* genes, whereas no *CsHSP20* genes were found on CsB09, CsA12, or CsB18 (Fig. 7). However, there were more *CsHSP70* genes within subgenome B (13 *CsHSP* genes) than within subgenome A (9 *CsHSP* genes). The *CsHSP90* genes were located on only 6 chromosomes, among which the majority were distributed on CsB02 and CsA05. *CsHSFC* genes were located only on CsB02, CsA05 and CsA12, and eight chromosomes of *C. songorica* did not contain any *CsHSFA* genes (Additional file 1: Fig. S7). We then identified homologous gene pairs, and the results showed that 25 homologous gene pairs belonged to the *CsHSP* gene family, including 16, 2, and 3 homologous gene pairs between subgenome A and subgenome B, within subgenome A, and within subgenome B, respectively (Fig. 7). Totals of 15, 4 and 2 homologous gene pairs were identified in the *CsHSP20*, *CsHSP70* and *CsHSP90* families, respectively. In addition, there were 13, 2 and 2 homologous gene pairs in the *CsHSP20*, *CsHSP70* and *CsHSP9*0 families between subgenome A and subgenome B. We also identified 24 homologous gene pairs in the *CsHSF* gene family, among which 14, 4 and 6 belonged to the *CsHSFA*, *CsHSFB* and *CsHSFC* groups, respectively (Additional file 1: Fig. S7). Most of the homologous gene pairs presented a Ka/Ks ratio < 0.3, with the highest ratio recorded for *CsHSP20–48*-*CsHSP20–45* (Ka/Ks ratio = 1.03). A similar trend was found for homologous gene pairs in the *CsHSF* gene family (Additional file 11: Table S10).

### Evolutionary and phylogenetic relationships among rice and *C. songorica HSP* and *HSF* genes

**Fig. 8:**
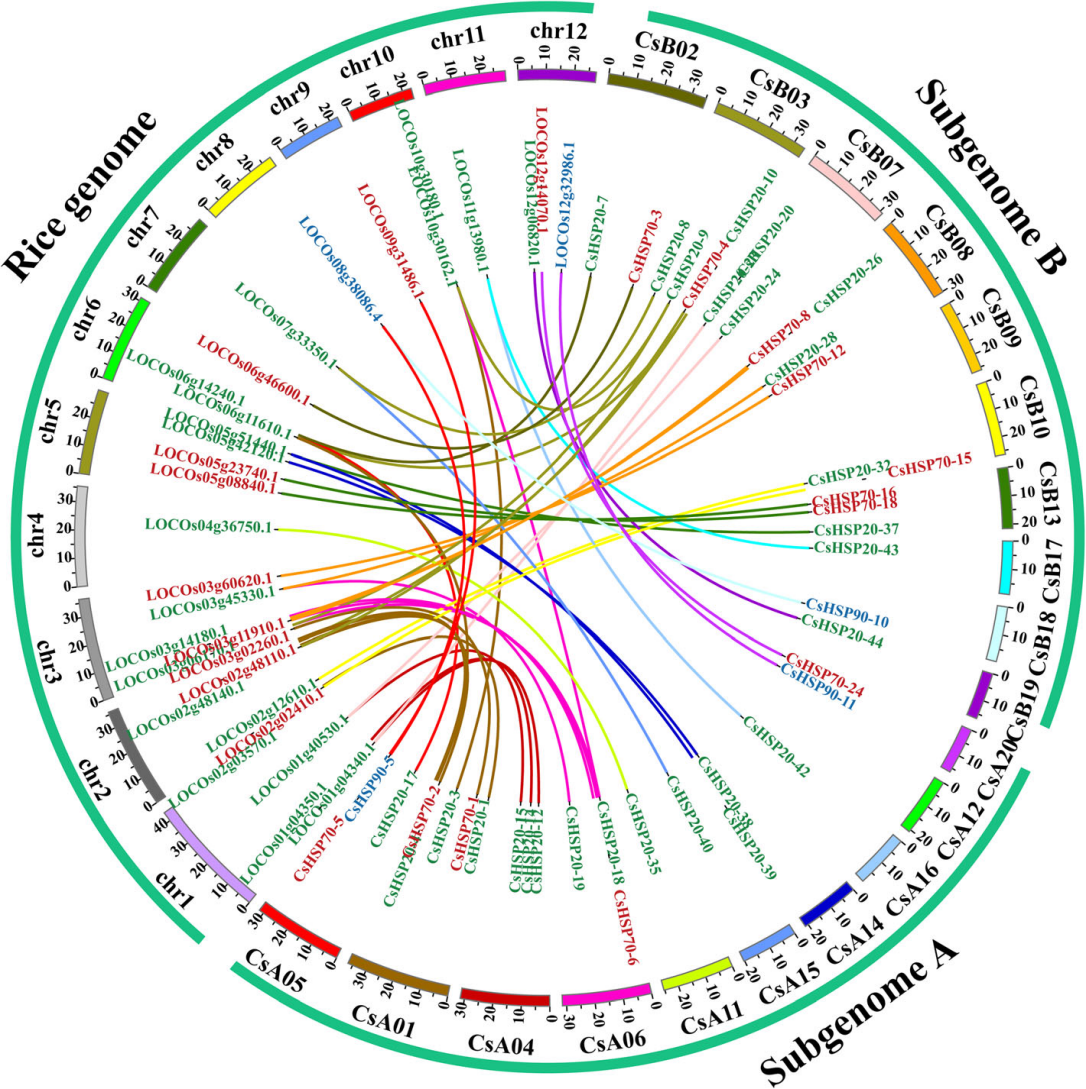
Distribution and synteny analysis of *HSP* genes between *C. songorica* and rice. The coloured links indicate *HSP* syntenic regions in the *C. songorica* and rice *HSP* gene families. Chr1 to Chr12 belong to rice, whereas subgenome A and subgenome B represent the 20 chromosomes of *C. songorica*

To deduce the evolutionary history, origin, and function of the *CsHSP* and *CsHSF* genes effectively, we performed a comparative genomic analysis between rice and *C. songorica*. Rice is one of the best-studied model plant species and is closely phylogenetically related to *C. songorica*. A synteny map of the rice and *C. songorica* genomes was constructed, revealing 49 and 22 orthologous gene pairs between the *HSP* and *HSF* genes, respectively (Fig. 8; Additional file 1: Fig. S8; Additional file 12: Table S11). Among these genes, 34, 12, 3, 15, 4 and 3 orthologous gene pairs were identified from the *HSP20*, *HSP70*, *HSP90*, *HSFA*, *HSFB* and *HSFC* groups, respectively. Notably, 9, 7, 1, and 1 orthologous gene pairs (one to one) were identified in the *HSP20*, *HSP70*, *HSP90*, and *HSFB* groups, respectively. We also found that one *CsHSP* and *CsHSF* gene each corresponded to multiple rice genes (Fig. 8; Additional file 1: Fig. S8). To explore the divergence of orthologous gene pairs between rice and *C. songorica*, the Ka/Ks ratios of the orthologous gene pairs were calculated on the basis of the information within the comparative synteny map. Most of the orthologous gene pairs presented Ka/Ks ratios < 0.3, with the highest ratio presented by *CsHSP20–18*-*LOC_Os03g06170.1* (Ka/Ks ratio = 0.48; Additional file 12: Table S11). A similar trend was found for the homologous gene pairs of the *CsHSF* gene family.

## Discussion

Heat stress is one of the key climatic parameters that affects plant growth and development. However, whole-transcriptome studies of *C. songorica* during HS are scarce. *C. songorica* is widely cultivated as ecological grass species in North-west China. To better understand the response of temperate grasses to HS, we measured both physiological parameters and large transcriptomic perturbations in heat-treated whole *C. songorica* seedlings for 0 to 72 h. HS can limit photosynthesis, causing a significantly decrease in the RWC. The effects of HS on plant photosynthesis can be evaluated by measuring chlorophyll fluorescence [31]. In *C. songorica*, the Fv/Fm, Y(II) and rETRmax values significantly decreased in response to 6 h of HS, indicating that *C. songorica* photosynthesis was limited by rETRmax under those conditions (Additional file 1: Fig. S1). We found that most of the genes whose expression was downregulated were involved in the chloroplast and its structural components, including multiple cellular components, which included GO terms such as ‘chloroplast stroma’, ‘chloroplast envelope’, ‘chloroplast thylakoid membrane’ and ‘chloroplast thylakoid’. In the biological process category, most of the genes whose expression was downregulated were related to photosynthesis and chlorophyll, including GO terms such as ‘photosystem II assembly’, ‘chlorophyll biosynthetic process’, ‘photosynthesis, light reaction’ and ‘chloroplast organization’ (Fig. 2). These results indicated that decreases in photosynthesis pathways may contribute to improved heat resistance in *C. songorica*. It is widely accepted that Pro acts as a cellular osmolyte and can increase plant resistance by accelerating biosynthesis [32]. In addition, Pro participates in the maintenance of redox balance and the scavenging of reactive oxygen species (ROS) [33]. Notably, ‘proline metabolism’ was identified among the enriched KEGG pathways of the DEGs. The expression of the associated genes was upregulated in *C. songorica* roots and shoots under HS. In addition, the Pro content was significantly greater in *C. songorica* under HS than under control conditions. These results indicated that Pro plays a positive role in the *C. songorica* response to HS, which has been confirmed in various plant species, such as kentucky bluegrass and cotton [34, 35].

Because *C. songorica* is an allotetraploid plant species, we identified the distribution of DEGs between its two subgenomes. Interestingly, the DEGs were evenly distributed between the two subgenomes. Specifically, 6971 and 7172 DEGs were distributed within subgenome A and subgenome B, respectively (Fig. 1c). We also found that the number of upregulated and downregulated genes did not differ between the two subgenomes. For instance, 4255 and 4298 DEGs whose expression was downregulated were identified in subgenome A and subgenome B, respectively, in *C. songorica* shoots. These results suggested that the two subgenomes of *C. songorica* played an equal role in the response to HS.

TFs are among the most promising targets for the improvement of plant performance under abiotic stress [36]. Members of certain TF families, such as the HSF, NF-YA, NF-YB, NF-YC and bZIP families, are known to mediate the HS response in plants [37–40]. In the present study, the expression of 1692 TFs from 50 families was found to be regulated in *C. songorica* in response to HS (Table 1). In total, 23 HSF, 7 NF-YA, 7 NF-YB and 36 NF-YC TFs were identified in *C. songorica*. *HSFA1* has been predicted to directly regulate the expression levels of important heat-responsive TFs, such as *DREB2A*, *HSFA2*, *HSFA7* and *HSFB*s [18]. Here, we found that one, two and one putative *HSF* genes from *C. songorica* were homologous to the rice *HSFA1*, *HSFA2* and *HSFA9* genes, respectively.

**Fig. 9:**
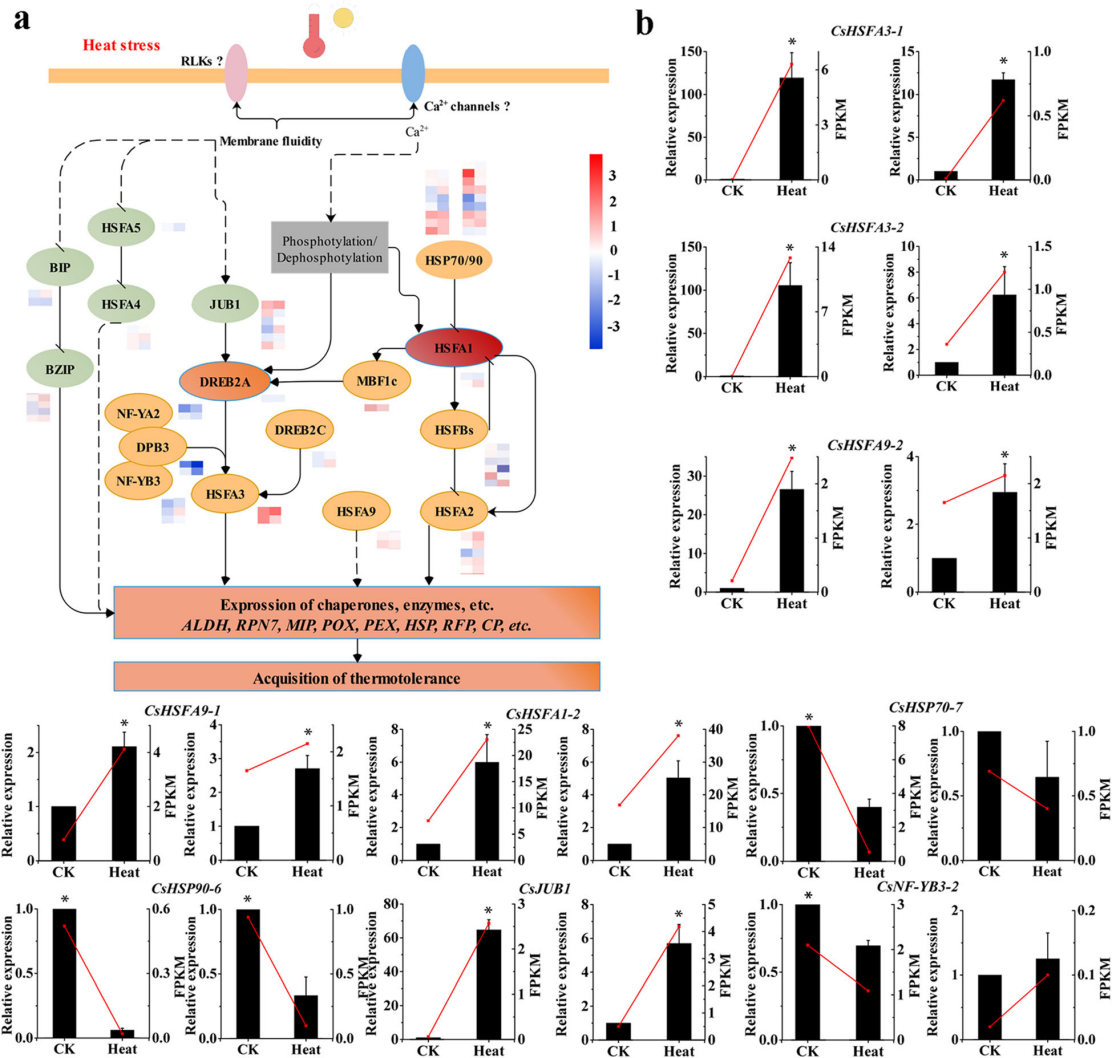
Regulatory network of the *C. songorica* response to HS. **a** Summary and proposed regulatory network of heat tolerance in *C. songorica*, *MBF1C*: *multiprotein bridging factor 1C*; *DPB3*: *DNA polymerase II subunit B3*; *NF-YA2*: *nuclear factor Y, subunit A2*; *NF-YB3*: *nuclear factor Y*, *subunit B3*; *JUB1*: *jungbrunnen1*. The heat map shows the log2(fold change) value of the genes. The left and right heat maps represent the shoots and roots, respectively. The solid lines denote links to confirm. **b** Relative gene expression levels of select genes. The data shown are the means of three biological replicates (*n* = 3), and the bar represents the standard error (SE) for each mean value. The left and right histograms represent the shoots and roots, respectively. * indicate a significant difference at *p* < 0.05 between control and heat treatment

Comparisons of transcriptomes between different species can provide information about the conservation of gene functions throughout evolution. In total, 216 orthologues were identified in all species; these genes constituted a core set of evolutionarily conserved genes associated with HS (Fig. 3). These conserved genes were largely involved in stress responses and activities within chloroplasts. KEGG pathway analysis revealed that the conserved genes were involved in ‘glutathione metabolism’, ‘endocytosis’ and ‘zeatin biosynthesis’. These pathways are known to be involved in responses to abiotic stress. Glutathione eliminates free radicals and protects the plant body [41]. Moreover, conserved genes that were associated with ‘glutathione metabolism’ and whose expression was downregulated under HS, which were homologous with the *GST* gene in Arabidopsis. Previous studies have shown that *AtGSTU17* was involved in glutathione metabolism under abiotic stress. When *AtGSTU17* was mutated, plants were more tolerant to drought and salt stresses compared with wild-type plants [42]. Endocytosis plays a core role in maintaining cell homeostasis, developmental processes, programmed cell death and stress tolerance [43, 44]. In the present study, 13 *PLD* genes involved in endocytosis and endosomal recycling pathways were identified. Furthermore, *PLD*s are involved in abscisic acid (ABA) signalling, drought, cold and salinity responses [45, 46]. Members of the *LRR-RLK* family, which constitutes one of the largest gene families, are involved in pathogen responses, development and abiotic stress responses in plants [46, 47]. In addition, RLKs and Ca^2+^ channels act as membrane-anchored proteins could sense the increasing membranes fluidity under HS (Fig. 9a) [48]. In total, we identified 23 conserved *LRR-RLK* genes within *C. songorica*. Expression of nineteen of these 23 *LRR-RLK* genes was downregulated in the shoots and roots under HS.

Based on the results of co-expression, some stress response genes were identified, which involved in posttranslational modification, signal transduction mechanisms, defense mechanisms, energy production and conversion, and inorganic ion transport and metabolism (Fig. 4). In model plants, *HSP* and *HSF* genes play a central role in HS and acquired thermotolerance [49]. *HSP* and *HSF* genes are also essential for normal growth and development in plants. Notably, the largest families of conserved DEGs identified belonged to the HSP superfamily, including 6 *HSP20*, 18 *HSP70* and 9 *HSP90* genes (Fig. 3). The *HSPs* were also identified to overlap genes of conserved genes and co-expression genes. We suggested *HSPs* play a core role in regulatory networks of *C. songorica* under HS, and confirmed our analysis results were reliable to reflect the regulatory networks of *C. songorica* under HS. Therefore, we identified the HSP and HSF superfamily members in the *C. songorica* genome. There are 84 *CsHSP* and 32 *CsHSF* genes in the *C. songorica* genome, including 48 *HSP20*, 25 *HSP70* and 11 *HSP90* genes (Fig. 5). We also found that 72.7% of the *HSP* genes were upregulated in *C. songorica* under HS. Compared with that which occurs in *Arabidopsis*, rice and *B. distachyon*, the results suggested that the *HSP* and *HSF* family of *C. songorica* has not tended to expand [22, 50, 51], which was confirmed by the Ka/Ks values of 21 homologous gene pairs of less than 0.3 in that species (Fig. 7; Additional file 1: Fig. S7). Furthermore, the *CsHSP* and *CsHSF* genes were evenly distributed between the two subgenomes of *C. songorica*. The Ka/Ks ratios of the orthologous gene pairs between rice and *C. songorica* also indicated that rice and *C. songorica* might have undergone purifying selection during their long evolutionary history.

## Conclusions

In this study, we performed conserved HS response genes identify, gene co-expression network analysis, and gene family identify by using RNA-seq data and genome information to reveal regulator network of *C. songorica* under HS. In this process, RLKs and Ca^2+^ involved in perceiving external signals in *C. songorica* under HS. *HSFA1* is first activated by the inhibition of *HSP70*/*HSP90* (Fig. 9a). The upregulation of *CsHSFA1* in turn plays a key role in activating the expression of downstream TFs such as *DREB2A*, *HSFA2* and *HSFB*s. *HSFB*s then inhibit the activity of *CsHSFA1* via a negative feedback loop (Fig. 9a). *DREB2A* regulates the expression of chaperones and enzymes in response to HS together with *NF-YA2*, *NF-YB3*, etc. Other pathways were also found to be important, including those involving *HSFA5*/*HSFA4* and *BIP* (Fig. 9a). The transcriptional regulatory network ultimately affects the expression of enzymes and chaperones via high expression levels of *CsHSFA3* and Cs*HSFA9*, which improves expression of chaperones and enzymes, and the thermotolerance and adaptation of *C. songorica* (Fig. 9b). This study revealed the regulatory network of *C. songorica* under HS on the basis of a comprehensive transcriptomic analysis and the identification of conserved genes. Our results provide comprehensive data for dissecting the molecular mechanism of HS responses in *C. songorica* and provide clues for thermotolerance improvement in grasses.

## Materials and methods

### Plant material and experimental treatments

Seeds of *C. songorica* were obtained from the *C. songorica* production field of Lanzhou University (103°08′N, 38°62′E), Minqin County, Gansu Province, China. The seeds of *C. songorica* were sown in a sand:vermiculite (1:1, v/v) mixture. The plates were maintained in a growth chamber supplemented with 150 μmol quanta m^− 2^ s^− 1^ irradiance under a photoperiod of 16/8 h (light/dark), 28/24 °C day/night temperatures, and 65% relative humidity [5]. Four-week-old seedlings were transplanted into plastic pots (height, 8 cm; top diameter, 8 cm; bottom diameter, 6 cm), with two plants per pot. Each pot contained 450 g of a sand:vermiculite (1:1, v/v) mixture. The experiment of temperature treatment was repeated in three growth chambers. After 5 weeks of cultivation, the temperature was increased to 40 °C to impose HS for 3 days. The shoots and roots were harvested after 0 h, 6 h, 12 h, 24 h, 36 h, 48 h and 72 h of HS, after which they were immediately frozen in liquid N_2_ and stored at − 80 °C for physiological and transcriptomic analyses.

### Characterization of physiological and photosynthesis traits

For the characterization of physiological and photosynthesis traits, eight seedlings were collected from the control group and the treatment group at the corresponding time points. The leaf materials of four seedlings were quickly harvested and used for the estimation of physiological traits, including MDA content, Pro content, and RWC. The MDA content and Pro content were determined according to the instructions provided by the manufacturer of the reagents (MDA-2-Y, PRO-1-Y; Komin, Suzhou, China; http://www.cominbio.com/). Afterward, the leaf samples were dried at 60 °C for 72 h to determine the RWC, which was calculated as follows: RWC% = (fresh weight-dry weight)/(saturated weight-dry weight)*100 [52]. An Imaging-PAM instrument (Waltz GmbH, Effeltrich, Germany) was used to measure Fv/Fm, Y(II) and rETR_max_ according to the manufacturer’s instructions [53]. Leaf temperature and soil temperature were measured with chlorophyll fluorescence system (Heinz Walz, Germany) and soil thermometer, respectively.

### Illumina RNA-Seq library construction and sequencing

Total RNA from each shoot and root was isolated via TRIzol reagent (Invitrogen, USA) according to the manufacturer’s instructions. Sequencing libraries were generated via a NEBNext® Ultra™ RNA Library Prep Kit for Illumina® (NEB, USA) following the manufacturer’s recommendations. M-MuLV reverse transcriptase (RNase H^−^) and random hexamer primers were used to synthesize first-stand cDNA. Second-stand synthesis was subsequently performed via DNA Polymerase I and RNase H. A total of 12 libraries (2 temperature treatment × 2 tissues × 3 replicates) were sequenced on the Illumina HiSeq platform, and 125 bp paired-end reads were generated. Low-quality reads, poly-N sequences from the raw data and reads containing adapters were removed to generate clean reads. Furthermore, HISAT2 software was used to map the clean reads to the *C. songorica* genome [4, 54], and StringTie (1.3.1) was used to quantify the gene expression levels according to the fragments per kilobase of transcript per million fragments (FPKM) method [55]. DEGs were analysed by the DESeq R package. The false discovery rate (FDR) was adjusted via the posterior probability of being differentially expressed (PPDE) method. Genes were considered differentially expressed when they presented a fold change ≥2 and an FDR ≤0.01. Three biological replicates were used for each sample. The GO annotation, GO enrichment and KEGG pathways were estimated via the BMK Cloud server (www.biocloud.net).

A heat map of gene expression was generated using OmicShare tools (http://www.omicshare.com/tools), and the Circos 0.69 program was used to show gene expression and gene locations on a genome-wide scale [56]. Last, a Venn diagram was generated by the jvenn website (http://jvenn.toulouse.inra.fr/app/example.html).

### Identification of TFs and conserved DEGs in response to HS in the Poaceae

A TF analysis was performed by the BMK Cloud sever, an online platform for data analysis (http://www.biocloud.net/). To identify subsets of HS-related DEGs shared between *C. songorica*, rice, *H. vulgare* and *B. distachyon* [57], select SRA data related to HS were downloaded from the NCBI database (PRJNA530826; PRJNA360513; PRJNA324116). The data analysis methods were the same as those described previously. OrthoMCL software V5 was used to identify orthologues with the default settings [58], and gene families were identified by Pfam (https://pfam.sanger.ac.uk/; e-value cut-off >1e^− 5^).

### Gene co-expression network analysis

The 59 RNA-seq data under abiotic stresses including data from this article were used to co-expression analysis [40]. According to the result from each sample regarding the DEGs expression quantity, we removed the expression quantity from all samples that were < 1 and expressions that barely changed (DEGs variance was < 0.75). R package WGCNA was used to construct gene co-expression networks [59]. The soft threshold (power) value with a signed R^2^ threshold > 0.85 was analysed by using PickSoftThreshhold function. We then used the automatic network construction function blockwiseModules to obtain weighted co-expression clusters, called modules, with the following settings for the calculation processes: power = 14, networkType = unsigned, corType = pearson, minModuleSize = 30, and mergeCutHeight = 0.25. A module that was significantly (*p* < 0.01) related to the heat stress samples is selected was selected to analysis in this study. Co-expression network was showed using Cytoscape (http://cytoscapeweb.cytoscape.org/).

### Identification and analysis of *HSP* and *HSF* gene families in the *C. songorica* genome

The protein and gene sequences of HSPs and HSFs derived from *Arabidopsis*, rice and *B. distachyon* were downloaded from Phytozome 12 (https://phytozome.jgi.doe.gov/pz/portal.html). These sequences served as queries for identifying the genes encoding the CsHSP and CsHSF proteins within the *C. songorica* genome via BLAST software (e-value cut-off>1e^− 5^). CD-HIT (http://weizhongli-lab.org/cdhit_suite/cgi-bin/index.cgi) tools were subsequently used to remove redundant sequences. The conserved domains within the candidate CsHSP20, CsHSP70, CsHSP90 and CsHSF proteins were further examined via Pfam (https://pfam.sanger.ac.uk/; e-value cut-off>1e^− 5^; PF00011; PF00012; PF02518; PF00447). Multiple sequence alignment of the HSP and HSF proteins of *C. songorica* was performed with ClustalX, with the default parameters. An evolutionary tree of the CsHSP and CsHSF proteins was then constructed with MEGA 7 software, with 1000 bootstrap replicates (http://www.megasoftware.net).

The HSP and HSF proteins of *C. songorica*, *Arabidopsis*, rice and *B. distachyon* were used for phylogenetic analysis (Additional file 13: Table S12). As such, their sequences were aligned with ClustalX software, with the default parameters. A maximum likelihood (ML)-based phylogenetic tree was constructed via IQTREE (1.6.11 version) software with 1000 bootstrap replicates [60]. The Shimodaira–Hasegawa-like approximate likelihood ratio test (SH-alRT branch test) was used to test the branch support, and the consensus tree topology was visualized with FigTree (version 1.4.3).

### Synteny analysis and chromosomal location of *HSP* and *HSF* genes

We used MCScanX software to search for gene pairs within the *C. songorica* and between the *C. songorica* and rice genomes (BLASTP: E < 1e-5, top 3 matches) [61]. A synteny map was generated via the Circos 0.69 program. The synonymous (Ks) and nonsynonymous (Ka) nucleotide substitutions were determined via ClustalW, PAL2NAL and the bio-pipeline yn00 program (https://github.com/tanghaibao/bio-pipeline/tree/master/synteny-pipeline).

### Quantitative real-time (RT) PCR

Total RNA was isolated from *C. songorica* shoots and roots after stress treatments via RNAiso reagent (TaKaRa, Dalian, China). For reverse transcription, first-strand cDNA was synthesized by a FastKing RT Kit (with gDNase; Tiangen, China). 2X TaqMan Fast qPCR Master Mix (Sangon, China) and a CFX96 instrument (Bio-Rad) were used with default settings to calculate the threshold cycle (CT) values. *CsGAPDH* was used as a reference gene. The specific primers used for the selected genes are shown in Additional file 14: Table S13. The relative expression levels were calculated by the comparative CT method [62]. All reactions were performed in triplicate.

## Supplementary information

**Additional file 1: Figure S1.** Effects of HS on the phenotype and physiological traits of *C. songorica* seedlings. (a) Phenotype of *C. songorica* at different time points under heat treatment. (b)-(g) Leaf RWC, MDA content, Pro content, Fv/Fm, rETRmax and quantum yield II. (h)-(i) Leaf temperature and soil temperature. Bars with different letters indicate significant differences at *P* ≤ 0.05 (Duncan’s test). **Figure S2.** GO enrichment of DEGs in the shoots and shoots of *C. songorica* under HS. *Red bar*, DEGs in the shoots; *green bar*, DEGs in the roots. **Figure S3.** Summary of identified TFs encoded by DEGs in *C. songorica* upon HS. **Figure S4.** KOG function classification of conserved DEGs; **Figure S5.** KEGG pathway enrichment of co-expression genes; **Figure S6.** Phylogenetic tree of HSF proteins in five species: *C. songorica* (yellow), rice (green), *B. distachyon* (blue), *Z. mays* (purple) and *Arabidopsis* (red). The tree was generated with IQTREE software via the ML method; **Figure S7.** Distribution and synteny analysis of *C. songorica HSF* genes. In the Fig., the 20 *C. songorica* chromosomes are shown as different coloured partial circles, and the chromosome numbers are indicated at the top of each bar. The coloured links indicate *HSF* syntenic regions in the *C. songorica HSF* gene family; **Figure S8.** Distribution and synteny analysis of *HSF* genes between *C. songorica* and rice. In the Fig., the 20 *C. songorica* chromosomes are shown as different coloured partial circles, and the chromosome numbers are indicated at the top of each bar. The coloured links indicate HSP syntenic regions in the *C. songorica* and rice HSF gene families. Chr1 to Chr12 belong to rice, whereas subgenome A and subgenome B represent the 20 chromosomes of *C. songorica*;

**Additional file 2: Table S1.** RNA-Seq data for twelve samples

**Additional file 3: Table S2.** Significant DEGs (FDR ≤ 0.01) were identified in at least one stress treatment, and their expression was regulated in the shoots and roots

**Additional file 4: Table S3.** List of over-represented significantly enriched GO terms shared between different combinations of organ/condition comparisons corresponding to the Venn diagram in Fig. 2a, b, c

**Additional file 5: Table S4.** Summary of DEGs that encode TFs identified in the transcriptome of *C. songorica* under HS

**Additional file 6: Table S5.** List of conserved heat-responsive genes shared across 4 Poaceae species and their expression patterns in *C. songorica*

**Additional file 7: Table S6.** The node and edge of co-expression network in *C. songorica* under HS

**Additional file 8: Table S7.** GO enrichment terms of co-expression genes of *C. songorica* under HS

**Additional file 9: Table S8.** The annotation and regulation of selected co-expression genes in *C. songorica* under HS

**Additional file 10: Table S9.** Characteristics of *HSP* and *HSF* genes in *C. songorica*;

**Additional file 11: Table S10.** Synteny blocks and Ka/Ks values of *HSP* and *HSF* genes within the *C. songorica* genome

**Additional file 12: Table S11.** Synteny blocks and Ka/Ks values of *HSP* and *HSF* genes between the *C. songorica* genome and the rice genome

**Additional file 13: Table S12.** The protein sequences of HSP and HSF from Arabidopsis, rice and *B. distachyon* and *C. songorica*

**Additional file 14: Table S13.** List of gene-specific primers used in this study.

## Data Availability

The datasets generated and during the current study are publicly available in the Sequence Read Archive (SRA) at NCBI (SRP218434; PRJNA530826; PRJNA360513; PRJNA324116) repository. The *C. songorica* genome are publicly available in the in the National Genomics Data Center (https://bigd.big.ac.cn/), under accession number GWHANUQ00000000 that is publicly accessible at https://bigd.big.ac.cn/gwh. The protein sequences of HSPs and HSFs derived from Arabidopsis, rice and *B. distachyon* were deposited in Additional file 14: Table S12.

## Abbreviation

HS: Heat stress
HSP: Heat-shock protein
HSF: Heat-shock transcription factor
DEGs: Differentially expressed genes
TFs: Transcription factors
GO: Gene ontology
RT-qPCR: Real-time quantitative PCR
