# Supplementary material for: Integrated analysis of co-expression, conserved genes and gene families reveal core regulatory network of heat stress response in Cleistogenes songorica, a xerophyte perennial desert plant
Source: BMC Genomics. 2020 Oct 16;21:715. doi: 10.1186/s12864-020-07122-8 (PMC7566159; doi:10.1186/s12864-020-07122-8)
Supplement: Supplementary file 1 — Additional file 1: Figure S1. Effects of HS on the phenotype and physiological traits of C. songorica seedlings. (a) Phenotype of C. songorica at different time points under heat treatment. (b)-(g) Leaf RWC, MDA content, Pro content, Fv/Fm, rETRmax and quantum yield II. (h)-(i) Leaf temperature and soil temperature. Bars with different letters indicate significant differences at P ≤ 0.05 (Duncan’s test). Figure S2. GO enrichment of DEGs in the shoots and shoots of C. songorica under HS. Red bar, DEGs in the shoots; green bar, DEGs in the roots. Figure S3. Summary of identified TFs encoded by DEGs in C. songorica upon HS. Figure S4. KOG function classification of conserved DEGs; Figure S5. KEGG pathway enrichment of co-expression genes; Figure S6. Phylogenetic tree of HSF proteins in five species: C. songorica (yellow), rice (green), B. distachyon (blue), Z. mays (purple) and Arabidopsis (red). The tree was generated with IQTREE software via the ML method; Figure S7. Distribution and synteny analysis of C. songorica HSF genes. In the Fig., the 20 C. songorica chromosomes are shown as different coloured partial circles, and the chromosome numbers are indicated at the top of each bar. The coloured links indicate HSF syntenic regions in the C. songorica HSF gene family; Figure S8. Distribution and synteny analysis of HSF genes between C. songorica and rice. In the Fig., the 20 C. songorica chromosomes are shown as different coloured partial circles, and the chromosome numbers are indicated at the top of each bar. The coloured links indicate HSP syntenic regions in the C. songorica and rice HSF gene families. Chr1 to Chr12 belong to rice, whereas subgenome A and subgenome B represent the 20 chromosomes of C. songorica; [file 12864_2020_7122_MOESM1_ESM.pdf]

Co-expression analysis and identification of conserved genes reveal regulatory network of heat stress response in *Cleistogenes songorica*, a xerophyte perennial desert plant

Qi Yan, Xifang Zong, Fan Wu, Jie Li, Tiantian Ma, Yufeng Zhao, Qian Ma, Penglei Wang, Yanrong Wang & Jiyu Zhang*

State Key Laboratory of Grassland Agro-ecosystems, Key Laboratory of Grassland Livestock Industry Innovation; Engineering Research Center of Grassland Industry, Ministry of Education, College of Pastoral Agriculture Science and Technology, Lanzhou University; Lanzhou, 730020, P. R. China

*Correspondence author: Jiyu Zhang: zhangjy@lzu.edu.cn

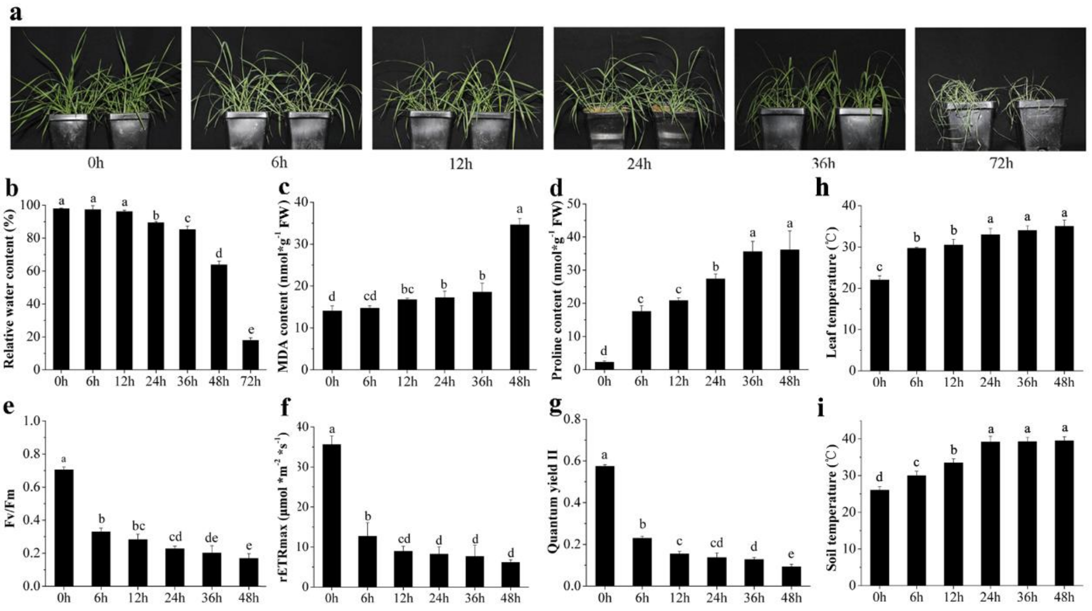

Fig. S1 Effects of HS on the phenotype and physiological traits of *C. songorica* seedlings. **(a)** Phenotype of *C. songorica* at different time points under heat treatment. **(b)-(g)** relative leaves water content, MDA content, Pro content, Fv/Fm, rETRmax and quantum yield II. The bars with different letters indicate significant differences at $p \leq 0.05$ (Duncan's test). **(h)-(i)** leaf temperature and soil temperature.

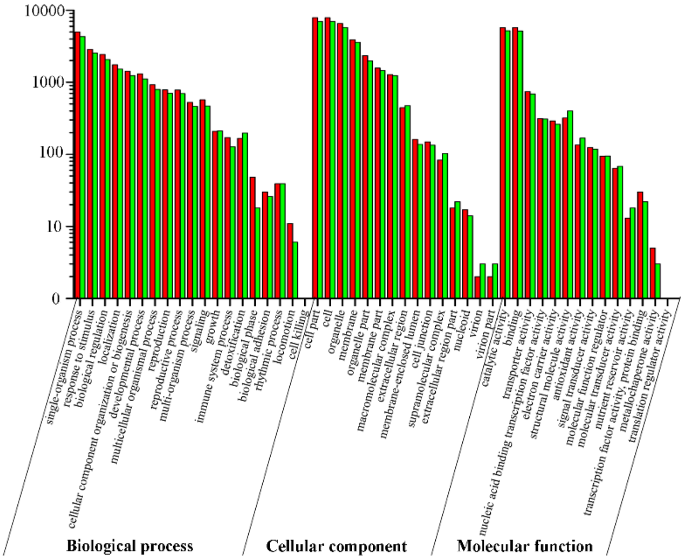

Fig. S2. GO enrichment of DEGs in the shoots and shoots of *C. songorica* under heat stress. *Red bar*, DEGs in the shoots; *green bar*, DEGs in the roots.

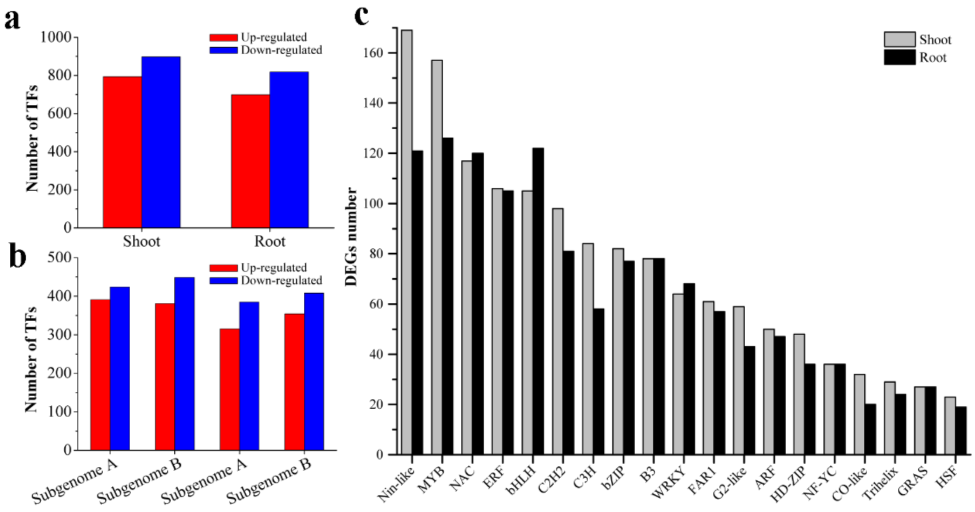

Fig. S3 Summary of identified TFs encoded by DEGs in *C. songorica* upon HS. (a) Number of upregulated and downregulated DEGs that encode TFs in the shoots and roots. **(b)** Distribution of upregulated and downregulated DEGs that encode TFs in the shoots and roots between the two subgenomes of *C. songorica*. **(c)** Distribution of DEGs that encode TFs responsive to HS in *C. songorica*. Only categories with more than 20 DEGs identified as encoding TFs are shown (Supplementary Table S4). *Black bar* DEGs in the shoots; *grey bar*, DEGs in the roots.

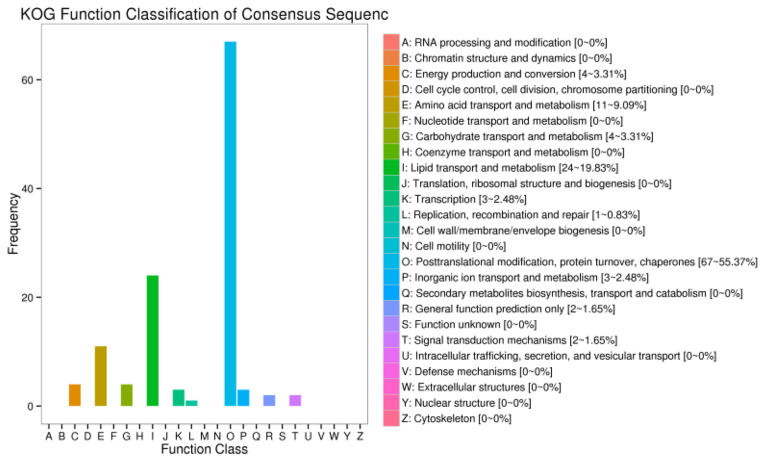

Fig. S4. KOG function classification of conserved DEGs.

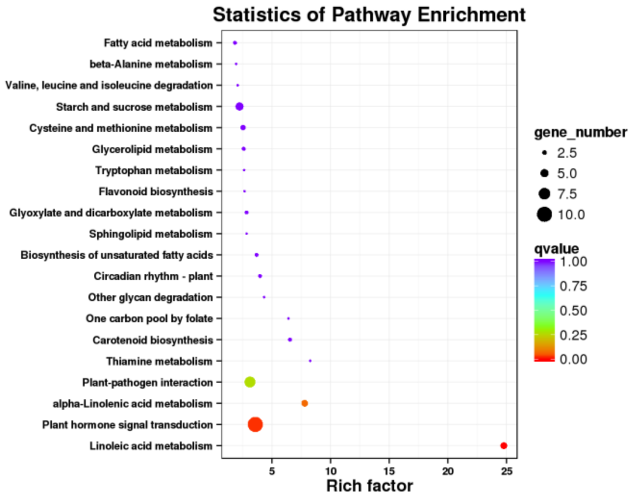

Fig. S5. KEGG pathway enrichment of co-expression genes.

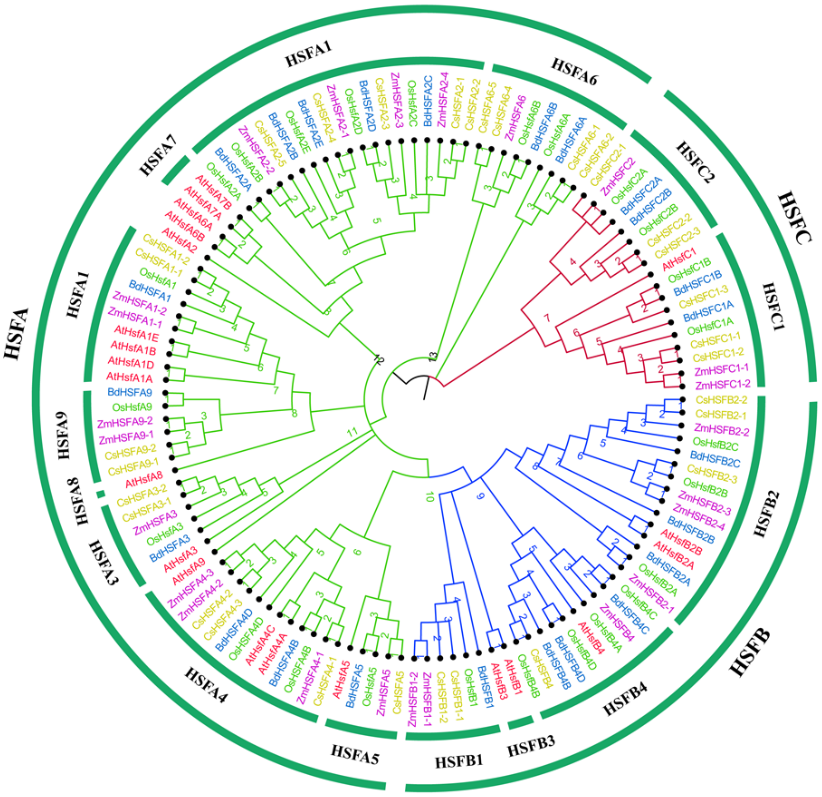

Fig. S6. Phylogenetic tree of HSF proteins in five species: *C. songorica* (yellow), rice (green), *B. distachyon* (blue), *Z. mays* (purple) and *Arabidopsis* (red). The tree was generated with IQTREE software via the ML method.

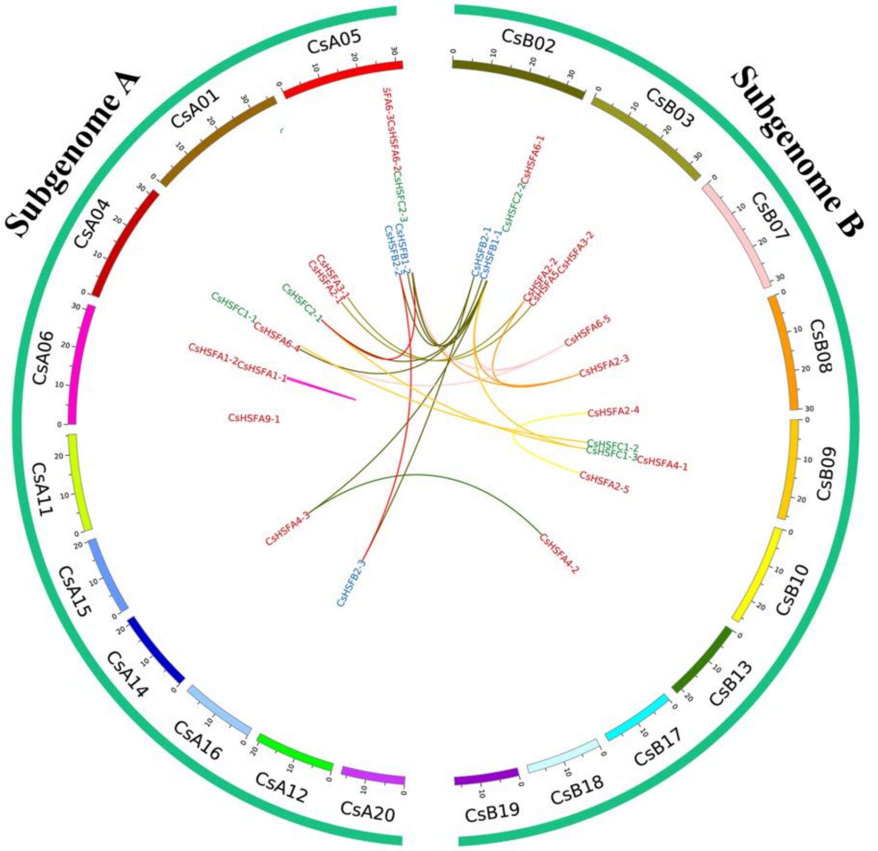

Fig. S7. Distribution and synteny analysis of *C. songorica HSF* genes. In the figure, the 20 *C. songorica* chromosomes are shown as different coloured partial circles, and the chromosome numbers are indicated at the top of each bar. The coloured links indicate *HSF* syntenic regions in the *C. songorica HSF* gene family.

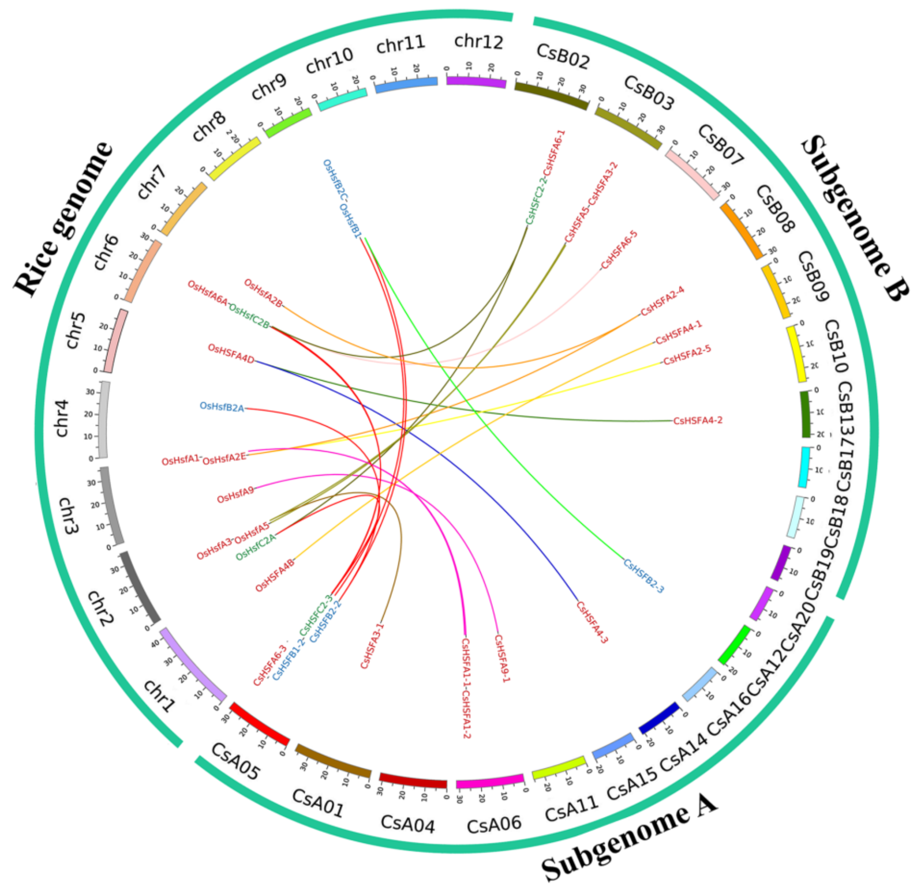

Fig. S8. Distribution and synteny analysis of *HSF* genes between *C. songorica* and rice. In the figure, the 20 *C. songorica* chromosomes are shown as different coloured partial circles, and the chromosome numbers are indicated at the top of each bar. The coloured links indicate HSP syntenic regions in the *C. songorica* and rice HSF gene families. Chr1 to Chr12 belong to rice, whereas subgenome A and subgenome B represent the 20 chromosomes of *C. songorica*.
